# Functionalized Ionic Liquids at Bacterial Membrane Interfaces: A Multiscale Perspective from Tensiometry, Microscopy, and Molecular Dynamics

**DOI:** 10.1021/acs.jpcb.5c08635

**Published:** 2026-03-25

**Authors:** Anita Wnętrzak, Joanna Feder-Kubis, Anna Chachaj-Brekiesz, Patrycja Dynarowicz-Latka, Jan Kobierski

**Affiliations:** † 37799 Jagiellonian University, Faculty of Chemistry, Gronostajowa 2, Kraków 30-387, Poland; ‡ 49567 Wrocław University of Science and Technology, Faculty of Chemistry, Wybrzeże Wyspiańskiego 27, Wrocław 50-370, Poland; § Technische Universität Dresden, Department of Inorganic Chemistry, Dresden 01069, Germany; ∥ Jagiellonian University Medical College, Faculty of Pharmacy, Medyczna 9, Kraków 30-688, Poland

## Abstract

Recently developed ionic liquids (ILs) are gaining attention for their antibacterial properties in the face of increasing resistance to conventional antibiotics. Their structural tunability enables the design of compounds that balance antimicrobial potency and acceptable toxicity. We investigated two monoterpene-based functionalized ionic liquids (FILs)—[(1*R*,2*S*,5*R*)-(−)-mentoxymethyl]­dimethyltetradecylammonium chloride and [(1*R*,2*S*,5*R*)-(−)-mentoxymethyl]­tetradecylimidazolium chloride—which differ in cationic core (ammonium vs imidazolium) but share an identical hydrophobic alkyl chain. Using a multiscale approach combining Langmuir monolayers, Brewster angle microscopy, atomic force microscopy, and molecular dynamics (MD) simulations, we examined their interactions with model bacterial membrane interfaces. Both FILs integrated readily into lipid monolayers, disrupted lipid packing, and altered interfacial organization, with pronounced effects in systems enriched in anionic lipids such as cardiolipin and POPG. Despite structural differences, the two FILs showed comparable antibacterial activity, notable given the typically higher toxicity of ammonium-based FILs. MD simulations revealed that electrostatic interactions dominate FIL–lipid association, with cationic headgroups binding preferentially to phosphate oxygens, while hydrogen bonding contributes minimally. The cations preferentially bind to phosphate oxygens of anionic lipids, explaining the enhanced activity against both Gram-positive and Gram-negative membrane models. The combined experimental–computational approach highlights key design principles for developing next-generation ILs with targeted activity at biological surfaces.

## Graphical abstract

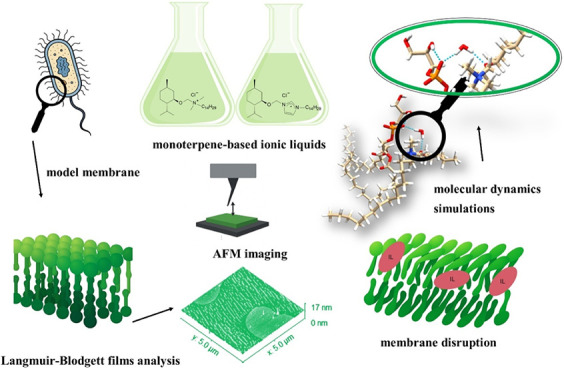

## 1 Introduction

According to the European Centre for Disease Control and Prevention (ECDC), approximately 30% of antibiotics are incorrectly prescribed by doctors.^1^ The result of excessive and inappropriate use of antibiotics and chemotherapeutics is the development of microbial resistance. For example, bacteria, through spontaneous mutations and horizontal gene transfer, acquire mechanisms to withstand drugs. They can also acquire resistance genes from other bacteria. Today, it is well established that bacteria have developed resistance to every class of antibiotics used clinically. Interestingly, in the case of penicillin, resistance was acquired even before the drug was used in clinical treatment.^2^ The increase in resistance to standard drugs is a global problem and concerns microorganisms in the hospital environment (approximately 70% of all infections) and those that occur in the community outside the hospital.^3,4^ ECDC data indicate that more than 700,000 infections caused by resistant bacteria are reported annually in Europe, resulting in more than 33,000 deaths from respiratory, bloodstream, and gastrointestinal infections.^1^ The problem of increasing resistance is further complicated by the fact that new drug discovery cannot keep up with the rate at which bacteria develop resistance. According to the World Health Organization (WHO) data, although the number of antibacterial drugs in clinical trials rose from 80 in 2021 to 97 in 2023, it is still insufficient.^5^ Moreover, many antibacterial agents, particularly hydrophilic ones, require the presence of channels formed by specific beta-barrel proteins, or porins, as well as other transporters, such as toxic oxygen radicals.^6–8^ Some antibiotics cannot use these pathways effectively to penetrate the outer membrane, which is why their action is ineffective. For example, vancomycin cannot penetrate the outer membrane and therefore is inefficient against Gram-negative bacteria.^7^ Therefore, there is an urgent need to advance active compounds with a broad spectrum of antibacterial activity. Ionic liquids (ILs), composed entirely of ions and notable for their highly designable physicochemical properties, which translate into tunable applications, may be promising in this field.^9–11^ The antibacterial properties of ILs can be tailored through rational structural design involving the selection of specific molecular components—such as the type of the amine core, the type of the anion, and the characteristics of the hydrophobic chain (its length and nature: alkyl, aryl, alkyl–aryl, or substituted)—as well as the introduction of task-specific functional groups mostly within the cationic moiety.^9,12^ Following this design strategy, several ILs have already been reported to display activity against antibiotic-resistant pathogens.^9,13,14^ For example, the effectiveness of several ILs against *Escherichia coli*, a Gram-negative bacteria responsible for infections primarily in the hospital environment, was demonstrated.^14^ In refs 14 and 15, the authors showed that the antibacterial effect depends on the length of the alkyl chain connected to the imidazolium cation. Interestingly, the application of the trihexyltetradecylphosphonium cation decreased the antibacterial activity of ILs but significantly reduced the toxicity to mammalian cells. Therefore, the appropriate selection of structural elements can introduce new therapeutic properties to existing popular drugs, potentially expanding the scope of their applications. Such an “improved” activity was demonstrated for ILs based on anions derived from standard antibiotics: ampicillin,^16^ penicillin G, and amoxicillin.^17^ In the case of imidazolium-based ILs, namely 1-alkyl-3-methylimidazolium chlorides, antibacterial activity was associated with disrupting the cytoplasmic membrane of *Staphylococcus aureus* (Gram-positive), leading to increased permeability and leakage of cytoplasmic contents.^15^ Hu et al. further demonstrated that an alkyl-based IL with a dodecyl chain, [C_12_MIM]­[Cl], induces reactive oxygen species generation, oxidative stress, and metabolic inhibition in *S. aureus*, followed by membrane collapse and cytoplasmic leakage. Electrostatic attraction between the positively charged imidazolium head and the negatively charged bacterial surface, combined with hydrophobic insertion of the alkyl chain into the phospholipid bilayer, was identified as the driving mechanism, which was also confirmed in vivo in a skin-abscess mouse model.^15^ The results are interesting because classic antibiotics used so far have a better effect on Gram-positive bacteria than Gram-negative ones.^18^

The antibacterial mode of action of ILs may be related to their activity at the membrane level.^9,19–21^ The incorporation of such ionic compounds into biological membranes alters the rheological properties of the membranes and the distribution of phospholipids, which consequently modifies membrane structural integrity and physiological function, and can cause irreversible damage.^9^ The directed action of ILs is further influenced by the biological membrane composition. It is assumed that bacterial membranes containing anionic lipids more readily attract ILs which, once incorporated, induce membrane lysis through strong electrostatic interactions.^9^ In turn, mammalian membranes containing zwitterionic phospholipids and cholesterol molecules constitute natural barriers to ILs. Additionally, as shown in ref 22, even when ILs are incorporated into the model of a mammalian membrane, they do not cause lipid reorientation, thus preventing host-membrane destabilization and reducing overall cytotoxicity.

A technique that can help to understand the membrane activity of ILs is the Langmuir monolayer technique.^23,24^ Biomimetic films at the liquid/gas interface serve as models of biological membranes, which can be employed to analyze the impact of bioactive substances,^25–30^ as well as the process of drug incorporation.^31,32^ Thermodynamic analysis of interactions between components in films allows identification of molecular targets on the film surface.^24^ Moreover, imaging techniques enable direct visualization of changes in the monolayer morphology.^33–36^ On the other hand, the involvement of molecular dynamics (MD) makes it possible to identify molecular-level mechanisms responsible for monolayer disruption (or stabilization) by ILs. MD simulations provide complementary insights into the nature of IL–lipid interactions, including the contributions of electrostatics, hydrogen bonding, and ion penetration. This approach has been successfully applied to study IL-induced changes in lipid packing and membrane perturbation.^37^ More broadly, MD has become a key tool for investigating IL nanostructuring, transport properties, and self-assembly in neat and mixed ILs.^38^ Together, Langmuir monolayers and MD simulations provide a multiscale experimental–computational framework for linking molecular interactions with macroscopic membrane behavior. Examples that illustrate the usefulness of Langmuir films in explaining the mechanism of action of drugs can be found in reviews and references therein.^24,25^

Notably, floating monolayers at the air–water interface formed from ILs have been scarcely investigated. Moreover, most studies probe the penetration of ILs into preformed lipid films rather than recording floating π–A isotherms of IL monolayers. In ref 12, cholesterol was reported to hinder the incorporation of ILs into lipid films and thus protect mammalian membranes against their destructive effects. This mechanism may be responsible for the selectivity of the IL antimicrobial action. In turn, the results presented in ref 20 showed that short-chain (*n* < 6) imidazolium ILs do not affect model cell membranes formed by DPPC, whereas chain elongation leads to the disruption of model cell membranes. The authors also observed that the destructive effect of ILs is stronger for DPPG films, a typical lipid of bacterial cell membranes.^20^ The destabilization of the membrane of Gram-positive bacteria due to the incorporation of ILs was also shown in ref 39. In turn, by using the Langmuir monolayer as a model of the *Escherichia coli* bacterial membrane (PE/PG (8:2) mixed system), it was shown that dialkylimidazolium-based-IL, [DMIM]­[TFSI], as a surface-active IL, induces membrane lysis in a mechanism regulated by electrostatic interactions.^14^

The main goal of our research is to examine whether and how two functionalized ILs (FILs) differing in the cationic part (ammonium vs imidazolium), namely [(1*R*,2*S*,5*R*)-(−)-mentoxymethyl]­dimethyltetradecylammonium chloride ([C_14_-Men-Am]­[Cl]) and [(1*R*,2*S*,5*R*)-(−)-mentoxymethyl]­tetradecylimidazolium chloride ([C_14_-Men-Im]­[Cl]), interact with biomimetic lipid monolayers representative of Gram-positive (Gram+) and Gram-negative (Gram−) bacterial membranes. In both FILs, functionalization consists of incorporating a bioderived monoterpene motif—(−)-menthol—known for its documented biological activity. The structures of both FILs and membrane lipids applied in experiments are shown in Scheme 1. The following model systems were selected to reproduce the lipid composition of the bacterial membranes: Gram-positive bacteria—DPPG/POPG/CL (1:1:2) and Gram-negative bacteria—POPE/DPPG/POPG (8:1.5:0.5).^40^ The bacterial cell wall was omitted in the present experiments because its peptidoglycan components cannot be examined by using the classic Langmuir monolayer technique. However, it is assumed that membrane destabilization constitutes a key step preceding bacterial cell wall lysis. Our monolayer experiments were supplemented with MD simulations to determine the orientation of the investigated FILs within the lipid environment and to identify the specific interactions responsible for IL–lipid binding, thereby distinguishing the effects of the different cationic cores. This combined experimental–computational approach enables a comparative molecular-level understanding of how ammonium- and imidazolium-based FILs interact with bacterial membranes, providing a foundation for rational antibacterial IL design.

**Scheme 1:**
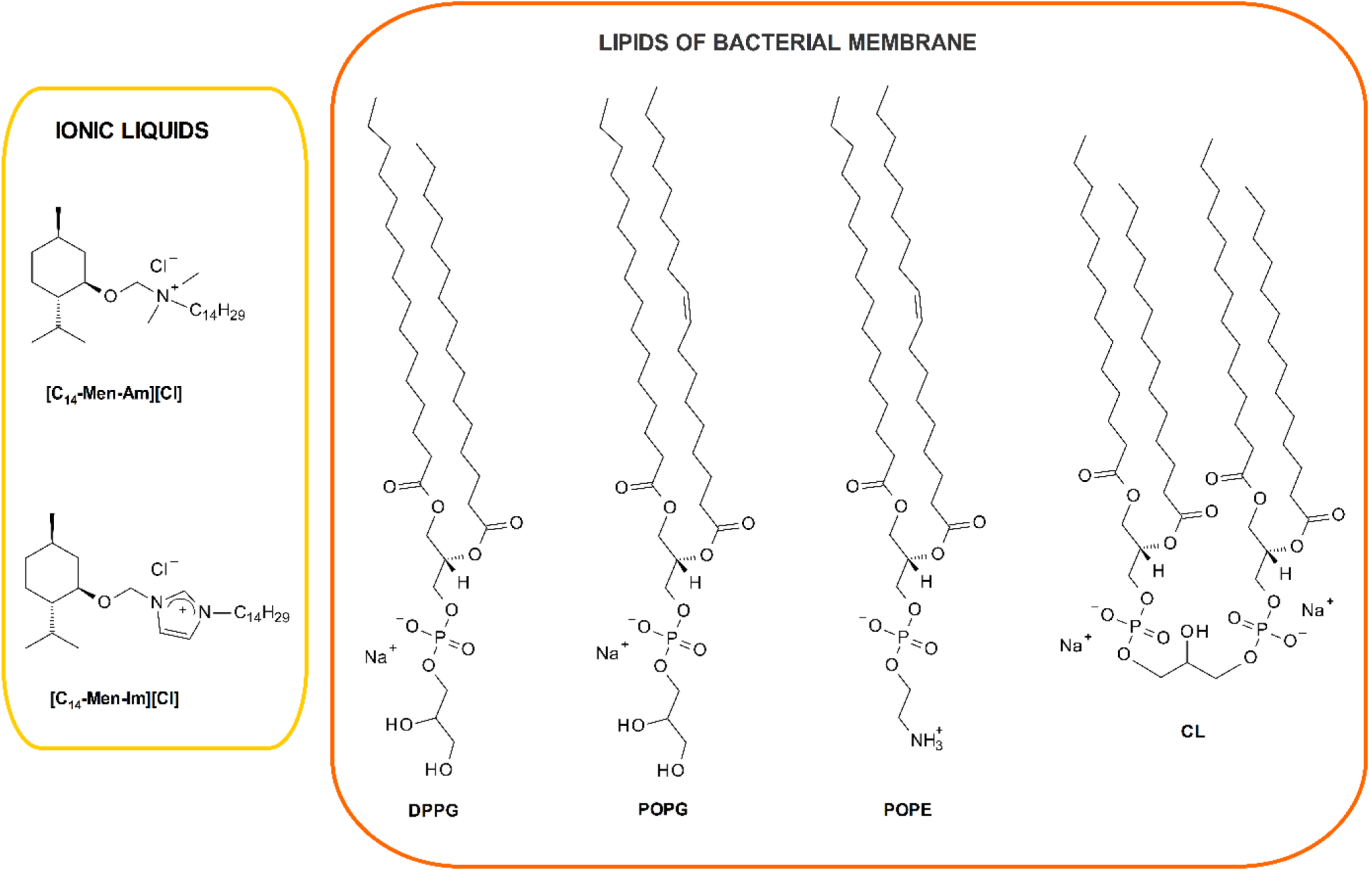
Structural Formulas of Functionalized Ionic Liquids [C_14_-Men-Am]­[Cl] and [C_14_-Men-Im]­[Cl] and Bacterial Membrane Lipids Used in Experiments

## 2 Methods

### 2.1 Materials

#### 2.1.1 Membrane Lipids

2.1.1

1′,3′-bis­[1,2-Dimyristoyl-*sn*-glycero-3-phospho]-glycerol (14:0 CL), 1,2-dipalmitoyl-*sn*-glycero-3-phospho-(1′-rac-glycerol) (DPPG), 1-palmitoyl-2-oleoyl-*sn*-glycero-3-phospho-(1′-rac-glycerol) (POPG), and 1-palmitoyl-2-oleoyl-*sn*-glycero-3-phosphoethanolamine (POPE) were purchased from Sigma–Aldrich (purity >99%) and used without further purification.

#### 2.1.2 FILs

2.1.2

The synthesis of the selected monoterpene-based FILs used in this study was reported in our earlier work: [(1*R*,2*S*,5*R*)-(−)-mentoxymethyl]­dimethyltetradecylammonium chloride ([C_14_-Men-Am]­[Cl])^12^ and [(1*R*,2*S*,5*R*)-(−)-mentoxymethyl]­tetradecylimidazolium chloride ([C_14_-Men-Im]­[Cl]).^22^

#### 2.1.3 Solvents and Subphase

2.1.3

Spectroscopic-grade chloroform (stabilized with ethanol; Sigma–Aldrich) was used for the stock solutions. Ultrapure water (HLP system) or its 0.1 M NaCl solution served as the subphase.

### 2.2 Experimental Section

#### 2.2.1 The Penetration and Adsorption Experiments

For the penetration and adsorption experiments, the investigated ILs were dissolved in ethanol to obtain stock solutions with concentrations of approximately 1.7 and 17 mg/mL. Measurements were performed in a Teflon cuvette specifically designed for surface-pressure measurements (with a capacity of 70 mL), featuring a round cavity and positioned over a magnetic stirrer. For adsorption experiments, the cuvette was filled with ultrapure water and, after several minutes of equilibration, the required volume of the corresponding FIL solution ([C_14_-Men-Am]­[Cl] or [C_14_-Men-Im]­[Cl]) was injected beneath the surface using a Hamilton microsyringe through the side slot of the cuvette. After the injection, the solution was homogenized with gentle magnetic stirring, and the resulting increase in surface pressure was measured for approximately 30 min by using the Wilhelmy method (accuracy of ±0.01 mN/m).

In penetration experiments, the chosen bacterial model membrane mixture was deposited dropwise onto the aqueous subphase using a microsyringe and allowed to stabilize for 4 min. The initial surface pressure of the spread monolayer was adjusted to approximately 33 mN/m. Subsequently, the appropriate volume of the respective FIL solution ([C_14_-Men-Am]­[Cl] or [C_14_-Men-Im]­[Cl]) was injected into the subphase through the side slot, and the change in the surface pressure (Δπ) was continuously monitored for about 30 min. Preliminary tests confirmed that ethanol alone had no significant effect on the surface pressure vs time curves (data not shown).

#### 2.2.2 Langmuir Monolayer Experiments

The investigated ionic compounds were dissolved in chloroform to prepare stock solutions with typical concentrations of 0.15–0.30 mg/mL. Mixed solutions of various compositions (FIL/lipid) were prepared by mixing the appropriate volumes of the respective stocks. Bacterial membranes were modeled as three-component Langmuir monolayers with the composition mentioned above. Langmuir films were formed in a Langmuir trough equipped with two movable barriers (KSV 2000; total area of 700 cm^2^) mounted on an antivibration table. Before each experiment, the trough was cleaned with ethanol and chloroform and the subphase surface was aspirated. Mixed-lipid solutions were spread dropwise onto the subphase using a Hamilton microsyringe (precision of ±2.5 μL). After 5 min allowed for solvent evaporation, the barriers were compressed at a rate of 20 cm^2^/min. Surface pressure (π) was measured with a Wilhelmy plate (ashless chromatography paper, Whatman Chr1) with an accuracy of 0.1 mN/m. The subphase temperature was maintained at 20 ± 0.1 °C using a Julabo circulator. Each isotherm was repeated at least three times to ensure reproducibility within ±2 Å^2^/molecule. Most analyses were performed at a pressure of 30 mN/m, corresponding to the so-called monolayer–bilayer correspondence,^24,41^ and, for comparative purposes, at low pressure (5 mN/m).

#### 2.2.3 Langmuir Monolayer Imaging at the Air–Water Interface

The morphology of the mixed Langmuir monolayers was investigated by using a Brewster angle microscope (Accurion GmbH, ultraBAM) equipped with a 50 mW laser (emitting *p*-polarized light at a wavelength of 658 nm) integrated with a double-barrier Langmuir trough (KSV 2000). BAM images were recorded at 720 μm × 400 μm. The polarizer and analyzer were set to *p*-polarization, and the incoming laser light was limited to an angle of incidence of 53° (the Brewster angle for the air–water interface at 20 °C). The monolayer formation procedure was identical to that described above.

#### 2.2.4 Langmuir–Blodgett (LB) Transfer of Monolayers onto Mica and Imaging with Atomic Force Microscopy (AFM)

To image the nanoscale topography of surface films, selected mixed systems were transferred onto freshly cleaved mica using the double-barrier Langmuir–Blodgett trough (NIMA 612D; total area 600 cm^2^). Transfers were performed at π = 30 mN/m (corresponding to biologically relevant membrane tension^42^) by depositing a single Langmuir monolayer with a dipper speed of 3 mm/min. Topography was acquired by atomic force microscopy (Nanosurf NaioAFM) in tapping mode (AC mode). Scan areas of 5 × 5 μm^2^ were recorded using Multi75Al-G probes (force constant k ≈ 3 N/m, resonance frequency f_0_ ≈ 75 kHz, cantilever length ≈ 225 μm). AFM images were obtained using Gwyddion software (version 2.58).

### 2.3 Molecular Dynamics

The molecular systems used in the simulations were constructed using Packmol.^43^ Lipids were parametrized with the Lipid21 force field,^44^ while the TIP3P model was employed for water.^45^ Electrostatic potentials for [C_14_-Men-Im]­[Cl] and [C_14_-Men-Am]­[Cl] were calculated quantum mechanically using Gaussian 16.^46^ Atomic point charges were calculated in the Antechamber program^47^ using the RESP model.^48^ The missing parameters were taken from the GAFF2 force field.^49^ The resulting topologies were converted using ACPYPE^50,51^ to GROMACS (2024)^52–55^ format, in which all molecular dynamics simulations were subsequently carried out. The energy of the systems was minimized by using a two-stage procedure. First, the steepest descent algorithm was applied with an energy convergence criterion of 100 kJ/mol nm for up to 50,000 steps. This was followed by conjugate gradient minimization with a tighter convergence criterion of 50 kJ/(mol nm) for 5000 steps. Equilibration was performed in four sequential steps comprising NVT and NPT ensembles with position restraints, using a 2 fs time step and totaling 1 ns of simulation time. Production molecular dynamics simulations were performed in the NPγT ensemble using a 2 fs integration time step and periodic boundary conditions. A semi-isotropic pressure coupling scheme was applied using the Parrinello–Rahman barostat with a reference pressure corresponding to a target surface pressure. Temperature was maintained at 293 K by using the velocity-rescale thermostat. Each system was simulated for 400 ns, and the final 50 ns were used for analysis. The results were analyzed in the CPPTRAJ program.^56^ Hydrogen bonds were identified using the geometric criteria, i.e., a donor–acceptor distance ≤3.0 Å and a hydrogen–donor–acceptor angle ≥135°.

## 3 Results and Discussion

### 3.1 Adsorption and Penetration of FILs into Monolayers Mimicking Bacterial Membranes

To verify that the investigated FILs adsorb at the air/liquid interface and incorporate into model lipid films, we performed adsorption and penetration experiments. In the adsorption experiments, we continuously monitored the surface pressure before and after injecting the FILs into the ultrapure water subphase beneath the air/water interface.

As can be seen in Figure 1A and B, both ILs adsorb onto the air/water interface immediately after their injection into ultrapure water; the initial increase in surface pressure depends on the concentration of FILs in the subphase (suggesting a lack of aggregation of compounds at the water surface), and the magnitude of this increase in surface pressure is greater for [C_14_-Men-Im]­[Cl]. Over time, we observed a gradual decrease in surface pressure for [C_14_-Men-Am]­[Cl] at concentrations above 1 × 10^–7^ mol/L and for [C_14_-Men-Im]­[Cl] at concentrations below 1 × 10^–5^ mol/L. This behavior indicates initial interfacial adsorption followed by partial desorption and redistribution into the bulk aqueous phase. To overcome this issue, instead of ultrapure water, a subphase with increased ionic strength (such as a sodium chloride solution) can be used,^12,22^ ensuring film stabilization via charge screening and reduced electrostatic repulsion between cationic FIL headgroups.

**Figure 1:**
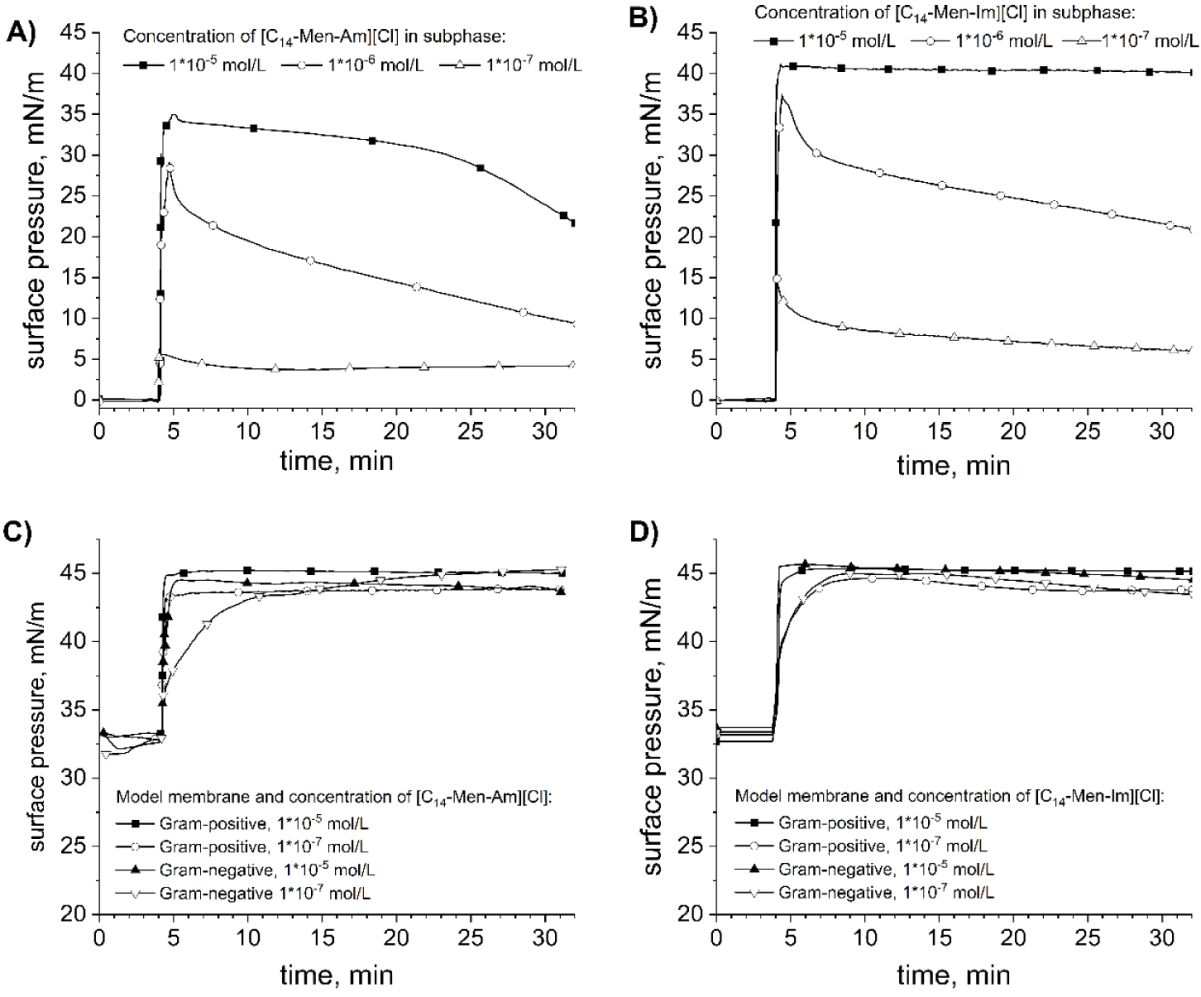
Adsorption of the investigated FILs at the air/water interface (A, B) and surface pressure vs time profiles for model membranes representing Gram-positive and Gram-negative bacteria before and after FIL injection into the subphase (C, D).

In addition to adsorption studies, the penetration experiments were performed by monitoring the surface pressure change before and after FIL injection beneath the preformed lipid monolayer, mimicking bacterial membranes. As illustrated in Figure 1C and D, both [C_14_-Men-Am]­[Cl] and [C_14_-Men-Im]­[Cl] incorporate into monolayers representing Gram-positive and Gram-negative bacterial membranes, which is supported by the stable increase in surface pressure from approximately 33 mN/m (before injection) to approximately 45 mN/m (12 min after injection), followed by a plateau maintained throughout the observation period. Interestingly, we found that the incorporation process was nearly immediate for higher FIL concentrations (1 × 10^–5^ and 1 × 10^–6^ mol/L), whereas for the lowest investigated concentration (1 × 10^–7^ mol/L) the surface pressure increased over about 10 min before reaching a plateau. It suggests that FIL molecules can insert into the tightly packed matrix formed by bacterial lipids; however, their further accumulation in the interfacial lattice is hampered due to the lack of enough space. This may lead to partial aggregation of excess FIL molecules in the interfacial region.

### 3.2 Interactions in Multicomponent Langmuir Monolayers Mimicking Bacterial Membranes

To investigate the effect of the studied FILs on bacterial membranes, we employed simplified artificial membrane models. Specifically, Gram-positive and Gram-negative membranes were represented as Langmuir monolayers with the lipid compositions described above (Section 2.2.1). The investigated FILs were added to both model membranes at a molar fraction of X = 0.25, keeping the proportions of lipids in the respective model systems constant. Additionally, for each π–A isotherm, the compression modulus (*C_s_* ^–1^) was calculated using the relation $C_{s}^{-1}=-A{\left(\frac{d\pi }{dA}\right)}_{T}$ formula, where *A* denotes the molecular area, and ${\left(\frac{d\pi }{dA}\right)}_{T}$ denotes the partial derivative at a constant temperature.^57^ π–A isotherms and the corresponding $C_{s}^{-1}$ profiles are shown in Figures 2 and 3. BAM images are shown in Supporting Information (Figures S1 and S2).

**Figure 2:**
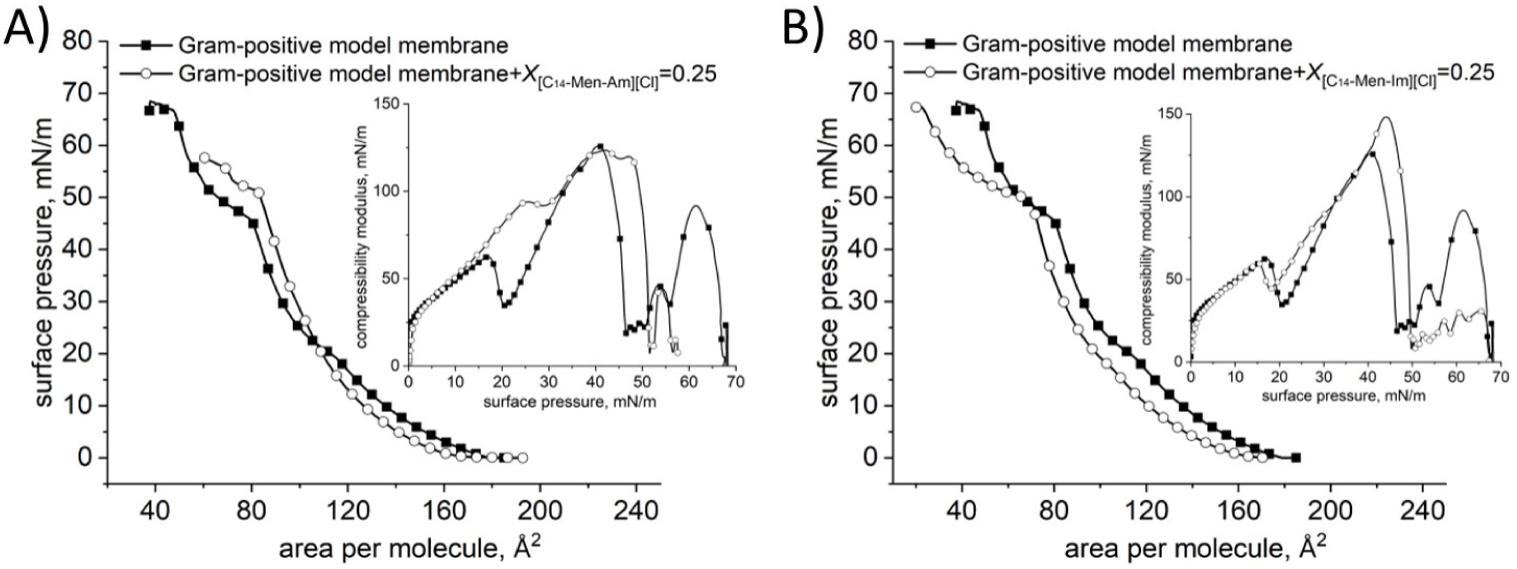
π–A isotherms of Langmuir monolayers mimicking Gram-positive bacterial membranes before and after A) [C_14_-Men-Am]­[Cl] and B) [C_14_-Men-Im]­[Cl] addition (X = 0.25). Insets: compressibility modulus *C_s_* ^–1^–π dependencies.

**Figure 3:**
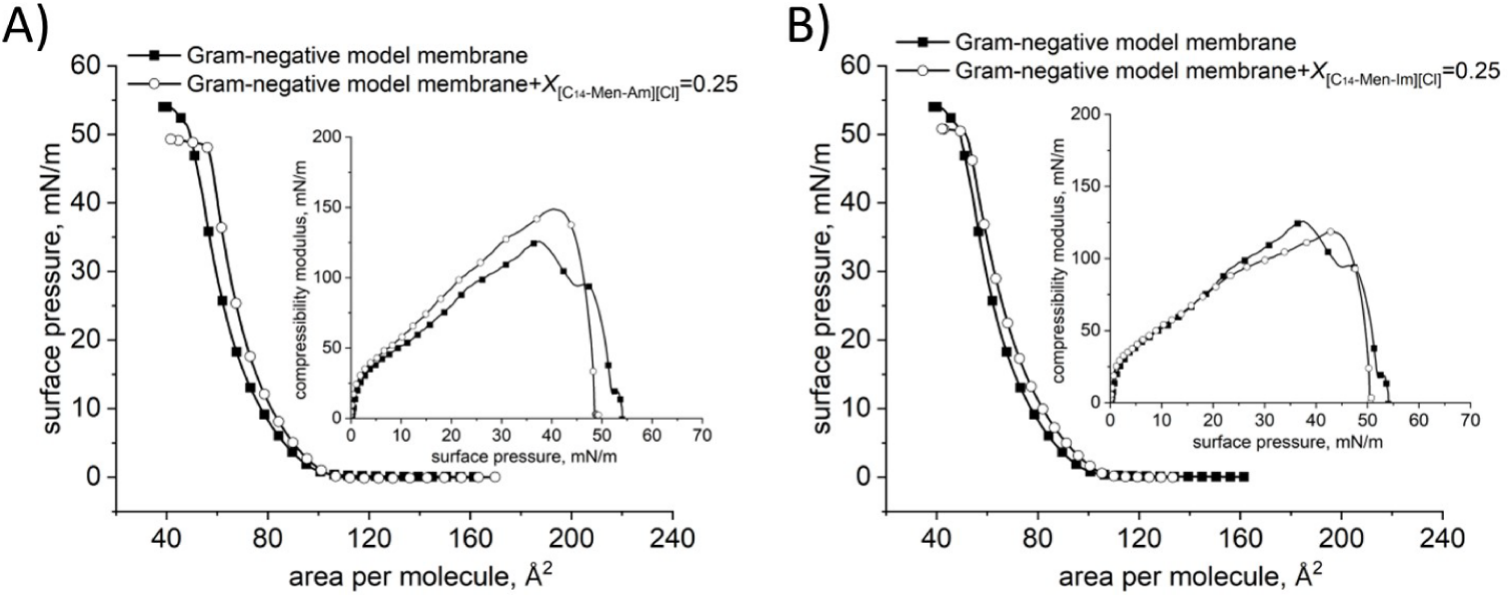
π–A isotherms of Langmuir monolayers mimicking Gram-negative bacterial membranes before and after A) [C_14_-Men-Am]­[Cl] and B) [C_14_-Men-Im]­[Cl] incorporation (X = 0.25). Insets: compressibility modulus *C_s_* ^–1^–π dependencies.

Based on the π–A isotherms and BAM images, we observed that the analyzed FILs modify the model membrane of Gram-positive bacteria more strongly than that of Gram-negative ones. For the Gram-positive membrane model, the addition of the IL shifts the π–A isotherm toward smaller molecular areas, whereas for the Gram-negative membrane model, the shift occurs in the opposite direction, toward slightly larger areas per molecule. However, these changes are relatively minor and, as indicated by the compressibility modulus values (see insets in Figures 2 and 3), they are not associated with a change in the physical state of the monolayers. Instead, they may reflect subtle molecular reorientation or rearrangement within the interfacial layer. As can be seen in the BAM images, the texture changes induced by FIL incorporation are clearly evident for the simplified Gram-positive membrane model, particularly in the presence of [C_14_-Men-Am]­[Cl]. In contrast, for the Gram-negative bacterial membrane-mimicking system, the effect of the FILs on the monolayer morphology appears negligible at the microscale, with the films remaining largely homogeneous. This can be explained by the high content of POPE in this model, for which (as well as for the analyzed FILs) the BAM images are rather homogeneous. To gain further insight into these subtle morphological effects, we performed nanoscale imaging of this system using AFM. For this purpose, the Langmuir films were transferred onto a solid substrate at a surface pressure of 30 mN/m and scanned with AFM. A comparable reorganization and domain formation upon FIL incorporation were previously observed for sterol-containing films,^12^ supporting the view that menthol-based ILs disturb membrane homogeneity. Representative AFM topographies of the studied ternary systems, together with the height profiles extracted along selected lines, are shown in Figure 4.

**Figure 4:**
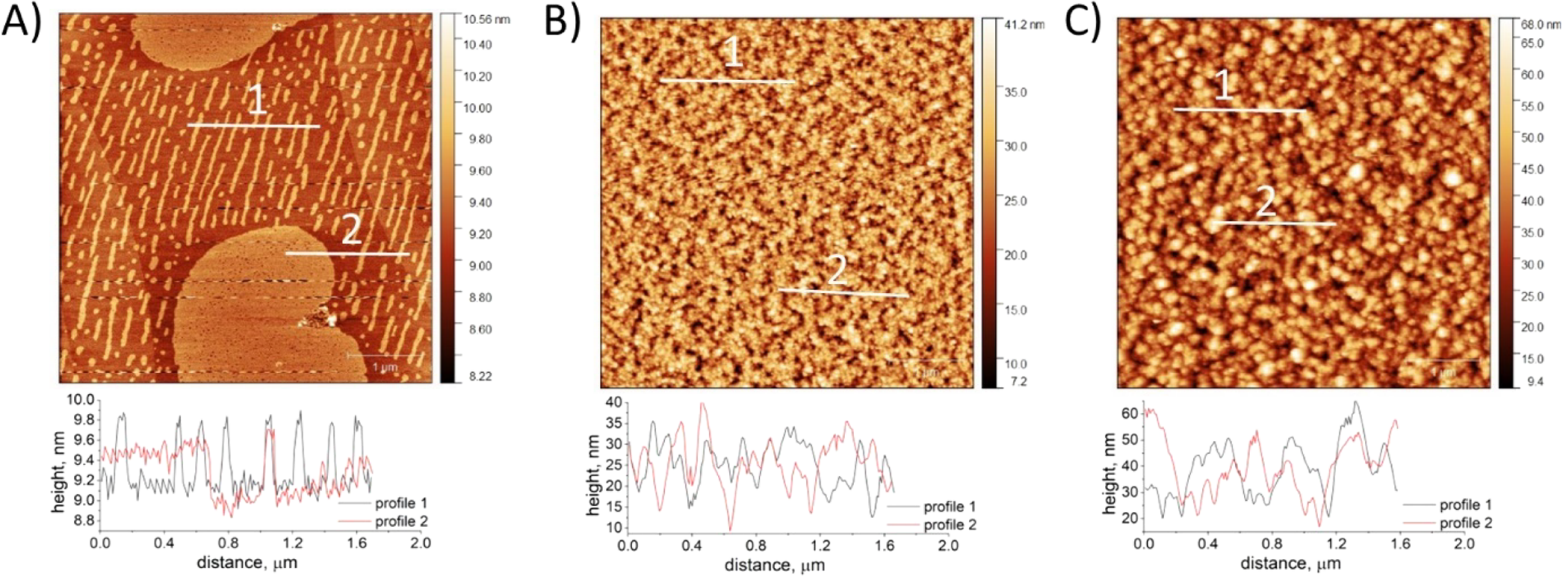
AFM topography images (5 μm × 5 μm) with profiles extracted along the white lines (1 and 2): A) Gram-negative membrane model, B) model with [C_14_-Men-Am]­[Cl], and C) model with [C_14_-Men-Im]­[Cl]. Single Langmuir monolayers were transferred onto mica at π = 30 mN/m.

AFM images reveal changes in the texture of an artificial Gram-negative bacteria-mimicking system upon FIL incorporation, which were not detected by BAM imaging. The model membrane of Gram-negative bacteria is characterized by domains with two different features: 1) elongated, approximately 0.1 μm wide and of various lengths, and 2) large (approximately oval) with a diameter of 2 μm. After incorporation of [C_14_-Men-Am]­[Cl] and [C_14_-Men-Im]­[Cl], the films became markedly rougher, indicating the formation of pronounced out-of-plane aggregates and three-dimensional surface features. Taking into account the resulting profiles, a significant increase in height difference (of approximately 15–35 nm in the case of [C_14_-Men-Am]­[Cl] and 20–60 nm for [C_14_-Men-Im]­[Cl]) between the three-dimensional surface features and the surroundings was observed. For comparison, AFM texture images for the artificial Gram-positive bacteria membrane-mimicking system are provided in the Supporting Information (Figure S3). In this case, also upon FIL incorporation, structural changes were observed: the previously flat sample became characterized by significant height differences between the phases, reaching up to 15 nm for [C_14_-Men-Am]­[Cl]. However, the aggregation of the molecules on the surface at the nanoscale is not as pronounced as that observed for the Gram-positive model. This is particularly noteworthy because, at the microscale (BAM) texture changes for the Gram-negative model were negligible. It should also be noted that Langmuir–Blodgett transfer can capture and preserve three-dimensional interfacial structures; therefore, the observed roughening and large step heights support FIL-induced reorganization and out-of-plane aggregation rather than purely two-dimensional phase separation. On the basis of the results obtained, it can be concluded that the tested FILs have a destructive effect on the simplified membrane-mimicking systems of Gram-positive and Gram-negative bacteria, leading to their reorganization at the micro- and nanoscale.

In the final stage, the interactions between FILs and the model membranes were evaluated from the excess Gibbs free energy of mixing ΔG^exc^ and the total Gibbs free energy of mixing $\Delta G_{tot}^{mix}$ . ΔG^exc^ was calculated based on formula: $N_{A}\int \left(A_{12}-A_{1}X_{1}-A_{2}X_{2}\right)d\pi$ ,^24^ where *A*_12_ denotes the mean molecular area per molecule in the mixed monolayer (model bacterial membrane + ILs), obtained directly from the experimental π–A isotherms; *A*_1_ represents the mean molecular area of the reference system, namely the phospholipid mixture constituting the artificial bacterial membrane model (DPPG/POPG/CL (1:1:2) or POPE/DPPG/POPG (8:1.5:0.5)); and *A*_2_ corresponds to the molecular area of the pure FIL measured at the same surface pressure at which *A*_12_ was determined. *X*_1_ and *X*_2_ are the mole fractions of the reference lipid system (*X*_1_ = 0.75) and the FIL (*X*_2_ = 0.25), respectively. On the other hand, $\Delta G_{tot}^{mix}$ is defined as $\Delta G_{tot}^{mix}=\Delta G^{exc}+\Delta G^{id}$ , where $\Delta G^{id}=RT\overset{N}{\underset{i=1}{\sum }}X_{i}\ln X_{i}$ and *R* is the gas constant, *X_i_* is the mole fraction of the reference lipid system (0.75 for the lipid mixture and 0.25 for FIL), and *T* is the absolute temperature.^24,58^ The π–A isotherms of pure FILs of the corresponding bacterial-membrane models served as reference systems. The results are presented in Figure 5.

**Figure 5:**
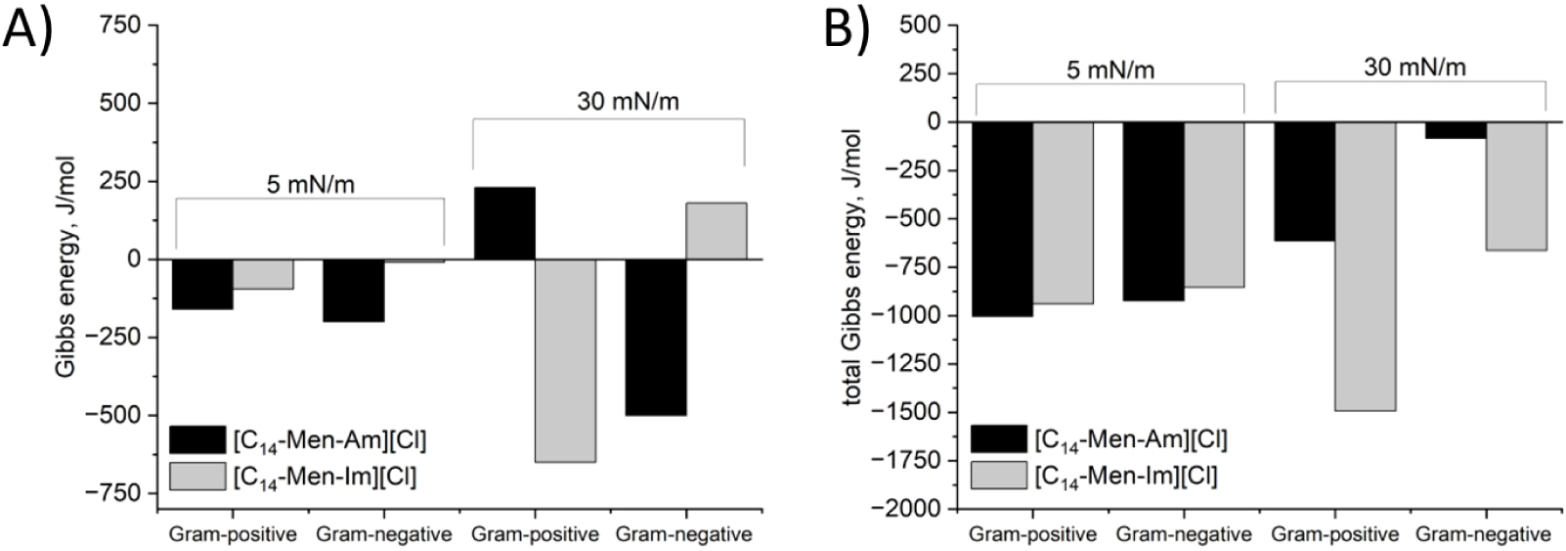
A) Excess Gibbs free energy of mixing ΔG^exc^ and B) total Gibbs free energy of mixing $\Delta G_{tot}^{mix}$ for lipid/ILs (X = 0.25) systems calculated in regions of low (5 mN/m) and high (30 mN/m) surface pressures.

The calculated interaction strength, expressed by the absolute value of ΔG^exc^, is strictly dependent on the surface pressure and increases upon compression. This trend is consistent with the decrease in molecular area and the progressive densification of the mixed monolayer under compression, a behavior widely observed for lipid and surfactant systems.^59^ The change in the strength of interactions at higher pressures is also evidence that ILs remain in the mixed layer and do not go into the water subphase. The continuous variation of ΔG^exc^ with surface pressure also confirms that FIL molecules remain embedded within the mixed film, without desorption into the aqueous subphase.^22^ Considering the monolayer–bilayer correspondence, the analysis was focused on the surface pressure of 30 mN/m, corresponding to the biological membrane packing density. As shown in Figure 5B, [C_14_-Men-Am]­[Cl] exhibits a larger magnitude of ΔG^exc^, indicating greater nonideality and a stronger perturbation of the mixing thermodynamics compared to [C_14_-Men-Im]­[Cl]. Importantly, ΔG^exc^ should be interpreted as a comparative thermodynamic signature rather than a direct quantitative metric of membrane destabilization in such multicomponent films, while BAM/AFM provides the primary evidence of structural disruption. When ΔG^exc^ is used in multicomponent systems, it should be taken into account that the calculated values reflect the combined contribution of many interactions, including FIL–lipid, lipid–lipid, and FIL–FIL effects. Thus, the lower stability observed for FIL/Gram-negative membrane-mimicking systems (Figure 5B) likely originates from the disruption of attractive interactions between POPE/POPG^60^ and POPE/DPPG^61^ pairs upon FIL incorporation. In turn, the lower $\Delta G_{tot}^{mix}$ values for the Gram-positive membrane-mimicking system can be attributed to the weakening of repulsive interactions between CL and DPPG^62^ following FIL incorporation. These diverse lipid–lipid and FIL–lipid interactions significantly influence the ΔG^exc^ values shown in Figure 5A. Their detailed molecular interpretation is provided in the subsequent section based on MD simulations. Regardless of the differences in ΔG^exc^, the morphological data (micro- and nanoscale) consistently indicate that FIL incorporation into both artificial bacterial membrane models (Gram-positive and Gram-negative) leads to partial structural disintegration. The incorporation of menthol-functionalized FILs promotes molecular reorganization and phase heterogeneity within the model bacterial membranes. Such modifications in interfacial interactions and film structure likely underpin the antibacterial activity of these compounds at the membrane level, in agreement with previous observations for related alkoxymethyl-based FILs.^39^

### 3.3 Analysis of Molecular Dynamics Simulations

To elucidate the interactions between ILs and simplified models of bacterial membranes of both Gram-negative and Gram-positive types, we performed molecular dynamics (MD) simulations of lipid bilayers. Each bilayer leaflet contained 192 lipid molecules. In the Gram-negative membrane-mimicking system, the composition per leaflet included 116 POPE, 21 DPPG, and 7 POPG molecules. The simplified Gram-positive membrane model consisted of 72 CL, 36 DPPG, and 36 POPG molecules per leaflet. In both systems, 48 IL molecules were incorporated into each leaflet, corresponding to a molar fraction of X_IL_ = 0.25. The bilayers were solvated with water in a ratio of 50 water molecules per lipid or IL molecule. To ensure charge neutrality, 56 Na^+^ and 96 Cl^–^ ions were added to the artificial Gram-negative system, and 432 Na^+^ along with 96 Cl^–^ ions were added to the artificial Gram-positive system.

For equilibrated systems, the surface pressure ranged from 33 to 34 mN/m, corresponding to area-per-lipid values of 64.12 Å^2^ and 64.50 Å^2^ for the Gram-negative membrane-mimicking system (experimental values: 62 Å^2^ and 62 Å^2^) with added [C_14_-Men-Am]­[Cl] and [C_14_-Men-Im], respectively, and 85.60 Å^2^ and 84.27 Å^2^ (experimental values: 85 Å^2^ and 85 Å^2^) for the Gram-positive membrane-mimicking system with added [C_14_-Men-Am]­[Cl] and [C_14_-Men-Im], respectively (Figure S4 and Table S1, Supporting Information).

#### 3.3.1 Electron Density Profiles

Analysis of electron density profiles revealed that both investigated FILs—[C_14_-Men-Am]­[Cl] and [C_14_-Men-Im]­[Cl]—integrate into both Gram-negative and Gram-positive membrane-mimicking systems (Figure 6). However, their localization and ways of interaction depend on the membrane composition. The FIL cations (as inferred from the positions of their N and O atoms) are predominantly located in the lipid headgroup region (∼15–20 Å from the bilayer center), indicating that they anchor at the membrane surface. Their charged headgroups are stabilized at this interface through electrostatic interactions with lipid phosphate groups, while the alkyl chains penetrate the hydrophobic core of the bilayer, reaching the bilayer center. Chloride anions (Cl^–^), introduced together with FILs to the simplified membrane models, remain in the aqueous phase: they accumulate near the artificial membrane surface but do not penetrate the lipid bilayer. Thus, Cl^–^ stays outside the membrane interface, while the FIL cations exhibit a strong affinity for anionic lipids—phosphatidylglycerol (PG) and cardiolipin (CL)—leading to pronounced accumulation of the cationic headgroups in the lipid headgroup region.

**Figure 6:**
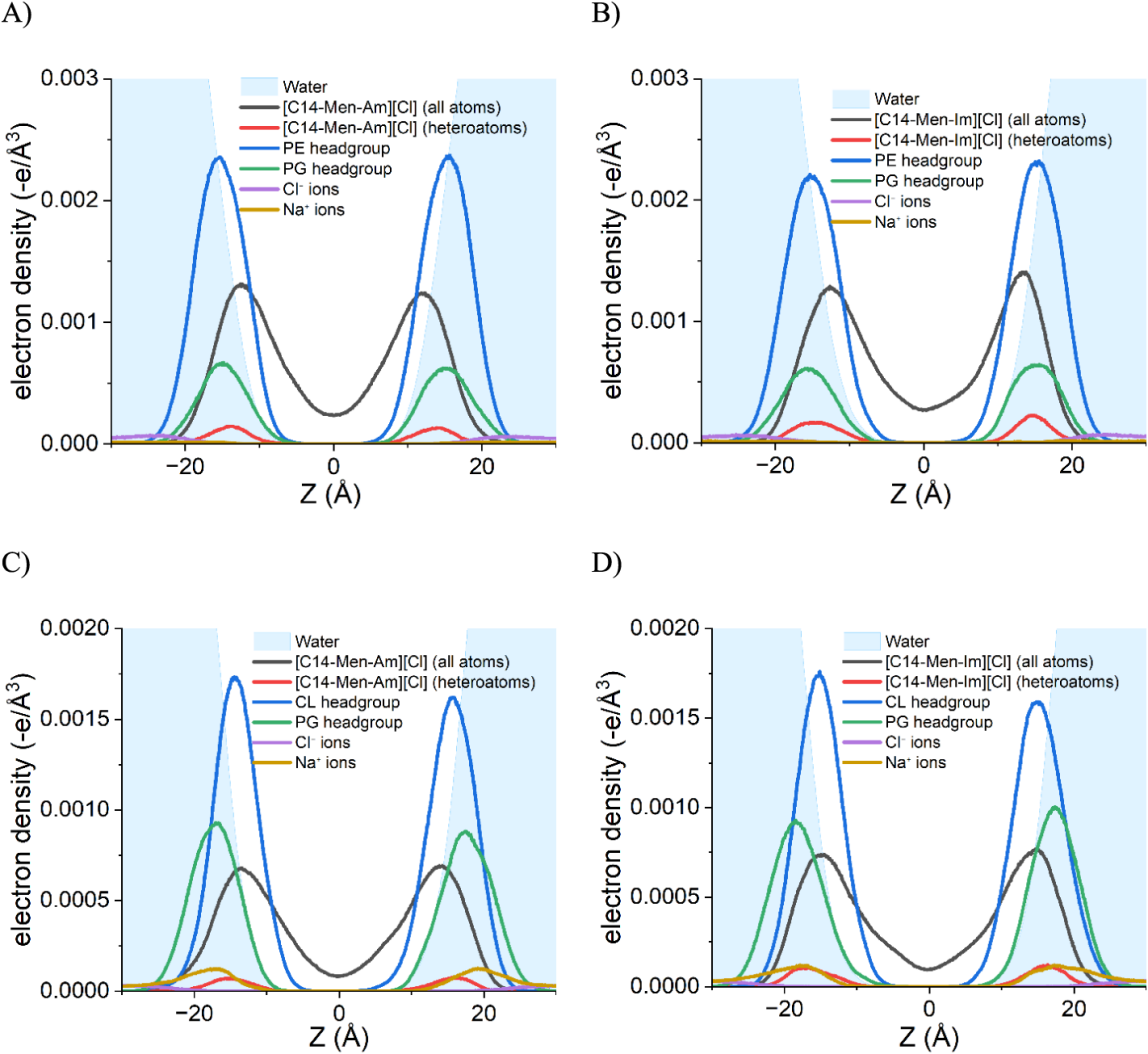
Electron density profile averages over analyzed time simulated in MD for Gram-negative (A and B) and Gram-positive (C and D) membrane-mimicking systems. FILs were added at a molar fraction of 0.25: [C_14_-Men-Am]­[Cl] in systems A and C, and [C_14_-Men-Im]­[Cl] in systems B and D.

The Na^+^ density profiles indicate that these ions remain present within the interfacial lipid region in the presence of the FILs. A detailed analysis of their spatial distribution relative to phosphate headgroups is provided in the following section.

#### 3.3.2 RDF Insights into Specific IL–Lipid Interactions

To better identify the specific atomic-level interactions between the ILs and various phospholipids, we computed detailed radial distribution functions (RDFs) for relevant pairs of atoms. We first examined RDFs for pairs consisting of an FIL heteroatom (either the cation’s nitrogen or oxygen present in the FIL structure) and a phospholipid oxygen or nitrogen in the lipid headgroups for each lipid type in the membrane models.

This analysis revealed clear differences in interaction patterns between the ammonium FIL [C_14_-Men-Am]­[Cl] and the imidazolium FIL [C_14_-Men-Im]­[Cl], as well as preferences for particular lipid types. One striking result is the difference in FIL–DPPG versus FIL–POPG interactions for the two FIL cations in the Gram-negative membrane-mimicking system (Figure 7). For [C_14_-Men-Am]­[Cl], the RDF amplitude is highest for interactions with DPPG, whereas for [C_14_-Men-Im]­[Cl] the highest RDF amplitude is observed with POPG—this is notable given that POPG’s mole fraction in the artificial Gram-negative membrane is about three times lower than DPPG’s in the Gram-positive membrane-mimicking system. In other words, [C_14_-Men-Am]­[Cl] shows a greater affinity for DPPG, while [C_14_-Men-Im]­[Cl] interacts more favorably with POPG. The imidazolium cation, with its aromatic ring and delocalized charge, can apparently adapt more readily to the more disordered or fluid environment created by unsaturated POPG, whereas the ammonium cation prefers the more ordered, tightly packed environment of saturated DPPG. This difference in selectivity may underline subtle variations in how each FIL disturbs different membrane models. In contrast to their robust interactions with PG, both FILs show weaker interaction with DPPE: the RDF for IL–PE atom pairs had the lowest amplitude despite PE being present in large excess. This confirms our earlier inference from density profiles that the FIL cations do not significantly interact with PE, instead showing a strong preference for negatively charged phosphatidylglycerols.

**Figure 7:**
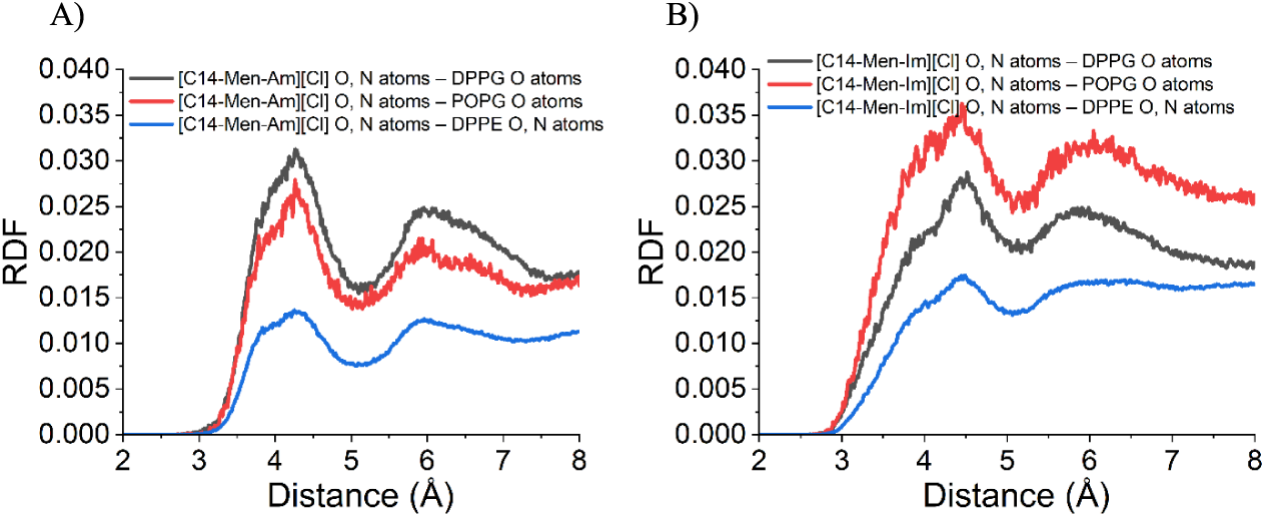
Radial distribution functions for pairs consisting of an FIL heteroatom: A) oxygen or nitrogen of [C_14_-Men-Am]­[Cl] and B) [C_14_-Men-Im]­[Cl] and a phospholipid oxygen or nitrogen in the lipid headgroups (DPPG—black, POPG—red, and DPPE—blue) for the Gram-negative membrane-mimicking system.

Hydrogen-bond analysis revealed no persistent hydrogen bonds between the FIL cations and lipid headgroups in the Gram-negative membrane-mimicking system. Only isolated, single-frame hydrogen-bonding events were detected: for [C_14_-Men-Am]­[Cl] with PG and for [C_14_-Men-Im]­[Cl] with both PG and PE. These correspond to very low occupancy and highly transient interactions rather than stable hydrogen bonding.

RDF analysis showed a first maximum at a distance of around 4 Å. This ∼4 Å peak is indicative of electrostatic attraction between charged atoms—specifically, the approach of the positively charged FIL nitrogen to the negatively charged nonesterified phosphate oxygen of a lipid. Such a distance (∼4–4.5 Å) could in principle also result from a water-bridged interaction (for example, a water molecule hydrogen-bonded between an FIL cation and a lipid phosphate, which yields an oxygen–oxygen distance of about 4.4 Å as observed in a DPPG–[C_14_-Men-Am], see Figure 8). However, those water-bridged configurations were found to be rare and transient in our simulations (occurring less than ∼7% of the sampled time, i.e., 3.5 ns of 50 ns). Therefore, we conclude that the dominant interactions at ∼4 Å are direct ion–ion or ion–dipole attractions between the FIL cation (nitrogen center) and the lipid phosphate oxygen rather than sustained water-mediated hydrogen bonds. The secondary, broader peak near ∼6 Å likely arises from the spatial arrangement of the second phosphate oxygen within the same headgroup, reflecting longer-range electrostatic interaction.

**Figure 8:**
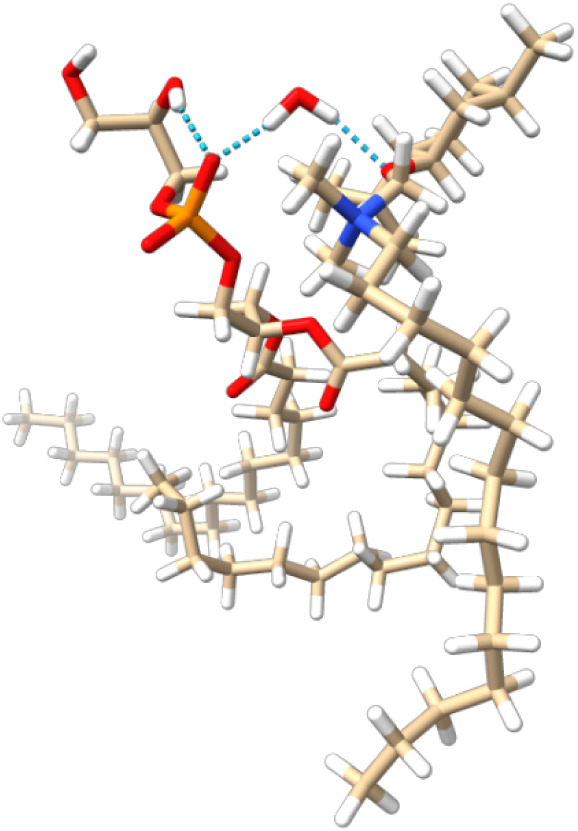
A water molecule forming a hydrogen bond with the oxygen atom of [C_14_-Men-Am]­[Cl] and the phosphate oxygen of phosphatidylglycerol—a water bridge captured in a molecular dynamics snapshot.

To pinpoint which specific atom pairs contribute most to these RDF peaks (and thus identify the most frequent contacts between FILs and lipids), we calculated RDFs for select individual atom pairings (see Figure S5, Supporting Information). For the Gram-negative membrane-mimicking system with [C_14_-Men-Am]­[Cl], the highest RDF amplitude was obtained for the pair of the FIL’s quaternary nitrogen (N^+^) and the nonesterified phosphate oxygen in the phospholipids (the oxygen atom in the phosphate group not involved in the ester linkage to glycerol). This confirms that the primary interaction is between the cationic head of the FIL and the phosphate group of the PG lipids. Interestingly, the two nonesterified phosphate oxygens in our PG lipids (DPPG or POPG) are not equivalent in their interactions due to the lipid’s molecular structure. One of the phosphate’s nonesterified oxygens (commonly denoted O33) is oriented toward the terminal glycerol and often participates in an intramolecular hydrogen bond with the glycerol’s hydroxyl, whereas the other phosphate oxygen (O34) is more exposed. Our analysis found that for [C_14_-Men-Am]­[Cl] interacting with DPPG, the IL’s nitrogen is more frequently in proximity to the glycerol-facing phosphate oxygen (O33). Additionally, when examining RDFs between the IL’s oxygen (from the cation’s menthol-based substituent) and the lipid’s terminal glycerol oxygens, we observed a small peak for the oxygen O35 (the hydroxyl oxygen in the middle of the terminal glycerol). This indicates that [C_14_-Men-Am]­[Cl] preferentially positions its cationic center near the junction of the phosphate group and the terminal glycerol moiety of DPPG, facilitating electrostatic interactions with the phosphate oxygen (O33) and occasional transient hydrogen bonding or bridging with the glycerol hydroxyl group (O35). In POPG, a different interaction pattern was observed: the [C_14_-Men-Am]­[Cl] nitrogen was found more often near the exposed phosphate oxygen (O34) rather than near the glycerol-associated side. Interactions involving the terminal glycerol hydroxyl of POPG were correspondingly less frequent. Overall, for the [C_14_-Men-Am]­[Cl] FIL in the Gram-negative membrane-mimicking system, the most common and stable interaction is the electrostatic attraction between the FIL’s N^+^ and the lipid’s phosphate oxygen, with water bridges or direct hydrogen bonds playing only a minor role.

We performed a similar specific-pair RDF analysis for [C_14_-Men-Im]­[Cl] in the Gram-negative membrane-mimicking system (see Figure S6, Supporting Information). The imidazolium cation has two ring nitrogen atoms (denoted N1 and N2), so we calculated RDFs for each of these nitrogens with lipid oxygens. The results showed that, much like the ammonium FIL, [C_14_-Men-Im]­[Cl]’s dominant interactions are with the nonesterified phosphate oxygens of PG lipids. Notably, the RDF peaks for the imidazolium nitrogens with POPG’s phosphate were higher in amplitude for POPG than for DPPG (consistent with the aforementioned preference of [C_14_-Men-Im] for POPG) and the RDF profiles for N1 and N2 were qualitatively similar, indicating that both nitrogens engage in similar interactions. However, there was a slight difference: for N1 (the ring nitrogen atom situated closer to menthol), the RDF peak was shifted to a shorter distance (∼3.7–3.8 Å) compared to N2 (which peaked around ∼4.5 Å). This implies that the N1 position of the imidazolium can approach the lipid phosphate slightly more closely than N2 can. The RDF for pairs involving the oxygen atom of [C_14_-Men-Im]­[Cl] and the terminal glycerol oxygen atoms of phosphatidylglycerol also exhibits a maximum at approximately 4.5 Å, which does not support the presence of stable direct hydrogen bonds.

Hydrogen-bond analysis revealed only isolated, single-frame hydrogen-bond occurrences between [C_14_-Men-Im]­[Cl] and the terminal glycerol of DPPG. In this system, we did detect the formation of water bridges, albeit in low numbers and with slightly increased persistence—lasting up to 28% of the analyzed simulation time. Overall, we conclude that the dominant interaction within this molecular pair is electrostatic attraction between the imidazolium ring of [C_14_-Men-Im]­[Cl] and the nonesterified phosphate oxygen atoms of phosphatidylglycerol (Figure 9).

**Figure 9:**
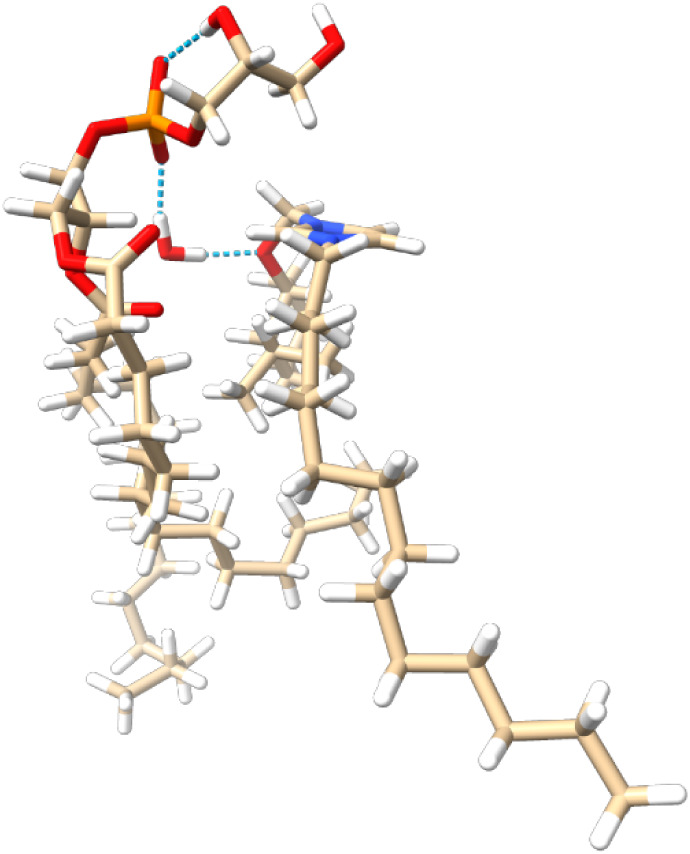
A water molecule forming a hydrogen bond with the oxygen atom of [C_14_-Men-Im]­[Cl] and the phosphate oxygen of phosphatidylglycerol—a water bridge captured in a molecular dynamics snapshot.

Turning to the Gram-positive membrane model, the RDF analyses revealed some differences in FIL behavior compared to those in the Gram-negative case. Considering first the broad FIL–lipid interactions (Figure 10): for [C_14_-Men-Am]­[Cl] in Gram-positive, the highest RDF peak by far was for the IL–cardiolipin pair (i.e., between FIL heteroatoms and any cardiolipin oxygen), reflecting the very strong attraction of the ammonium FIL to cardiolipin. The RDF peaks for IL–DPPG and IL–POPG pairs were comparatively lower, but between those two, DPPG showed a slightly higher peak than POPG. This indicates that in a Gram-positive environment with mixed anionic lipids, [C_14_-Men-Am]­[Cl] preferentially binds to cardiolipin and also has a greater affinity for DPPG over POPG. In contrast, [C_14_-Men-Im]­[Cl] in the Gram-positive membrane produced overall higher RDF amplitudes with all phospholipid types compared to [C_14_-Men-Am]­[Cl], suggesting that the imidazolium cation interacts more uniformly (and generally more strongly) with the anionic lipids present. The RDF values for [C_14_-Men-Im] with CL, DPPG, and POPG were all elevated and relatively comparable to each other (each higher than the corresponding values for [C_14_-Men-Am]), with the largest value for DPPG. Similarly to the previous systems, hydrogen-bond analysis indicated that interactions involving [C_14_-Men-Am]­[Cl] with PG or CL were negligible and occurred only as rare, single-frame contacts. In contrast, for [C_14_-Men-Im]­[Cl], hydrogen-bonding events with PG were observed more frequently, although they remained transient.

**Figure 10:**
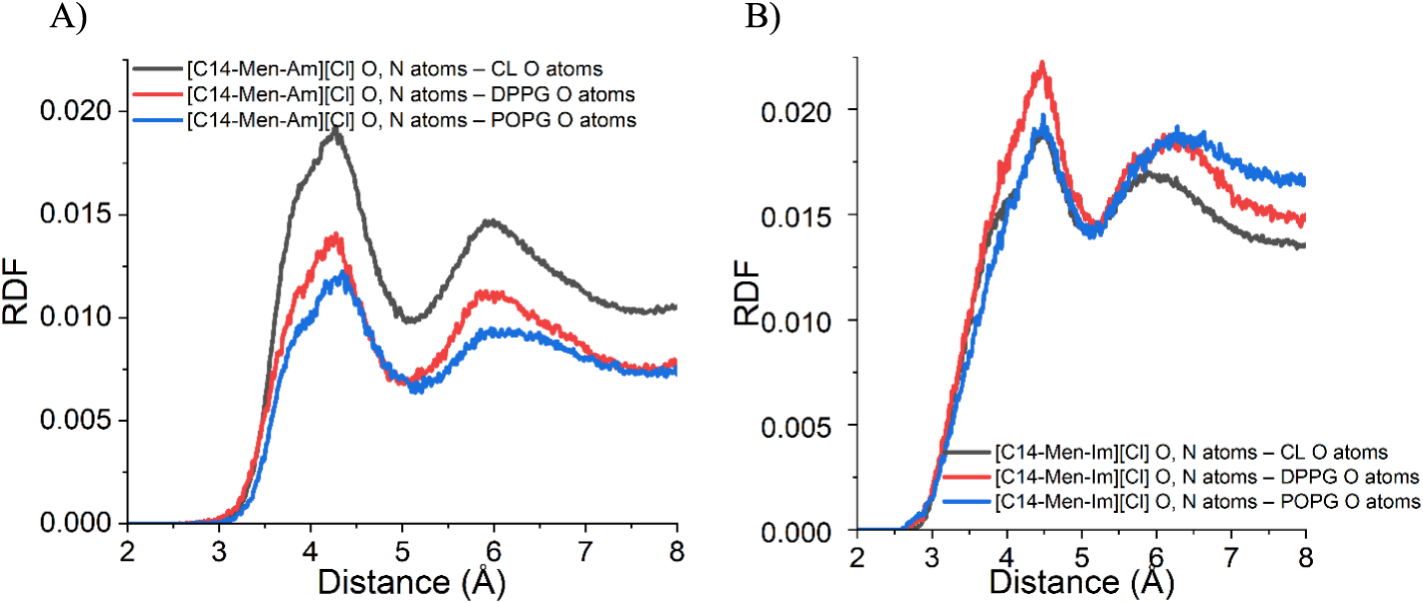
Radial distribution functions for pairs consisting of an IL heteroatom: A) oxygen or nitrogen of [C_14_-Men-Am]­[Cl] and B) [C_14_-Men-Im]­[Cl] and a phospholipid heteroatom (oxygen or nitrogen in the lipid headgroups of CL—black, DPPG—red, and POPG—blue) for the Gram-positive membrane-mimicking system.

A more detailed look at specific atom pairs in the Gram-positive membrane-mimicking system supports these observations. For [C_14_-Men-Am]­[Cl], the RDF for the pair of the IL’s N^+^ with DPPG’s nonesterified phosphate oxygen (again the glycerol-side oxygen, O33) had the highest peak and it was shifted to a short distance (around 3.5 Å), more so than any other pair—suggesting that [C_14_-Men-Am] often closely approaches DPPG’s phosphate (especially that O33 oxygen) in the Gram-positive membrane-mimicking system (see Figure S7, Supporting Information). The next highest RDF peaks were for the IL N^+^ with cardiolipin’s phosphate oxygens, which were also significant (and interestingly, the values were comparable for CL’s all phosphate oxygens, indicating that [C_14_-Men-Am] can bind to either oxygen on the cardiolipin headgroup with similar propensity). Interactions between [C_14_-Men-Am]’s N^+^ and POPG’s phosphate oxygens in the Gram-positive membrane-mimicking system were lower, reflecting the IL’s preference for the more highly charged or saturated lipids (CL and DPPG). Meanwhile, RDFs for the IL’s oxygen (menthol O) with the phosphate oxygens in the Gram-positive membrane-mimicking system showed a pattern analogous to the Gram-negative membrane-mimicking system: for DPPG, [C_14_-Men-Am]’s O interacted more with the glycerol-associated phosphate oxygen than the exposed one, whereas for POPG the difference was negligible and both oxygens saw similar, lower interaction.

For [C_14_-Men-Im]­[Cl] in the Gram-positive membrane-mimicking system, a hydrogen bond analysis showed the occurrence of interactions between [C_14_-Men-Im]­[Cl] and the O35 and O36 atoms in the terminal glycerol groups of DPPG and POPG (see Figure S8, Supporting Information); however, these interactions were transient. The longest-lived hydrogen bond between [C_14_-Men-Im]­[Cl] and DPPG persisted for approximately 9% of the analyzed simulation time (4.7 of 50 ns), while the POPG–[C_14_-Men-Im]­[Cl] hydrogen bond lasted for about 24% of the time, corresponding to 11.8 ns of 50 ns (Figure 11). In addition to hydrogen bonding, [C_14_-Men-Im]­[Cl] also showed strong electrostatic interactions with cardiolipin similar to [C_14_-Men-Am] (CL serving as a strong binding site for both cations), and its interactions with DPPG and POPG, while more balanced, still indicated a slight edge for DPPG in the Gram-positive membrane-mimicking system.

**Figure 11:**
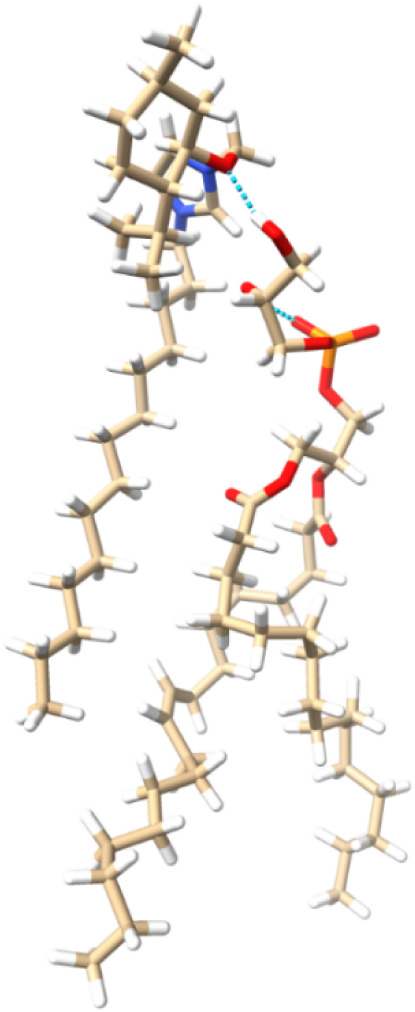
A snapshot from molecular dynamics simulation showing a hydrogen bond between the oxygen atom of [C_14_-Men-Im]­[Cl] and the hydroxyl group of the terminal glycerol moiety in POPG. An intramolecular hydrogen bond between the nonesterified phosphate oxygen (O33) and a glycerol hydroxyl is also observed.

To quantify possible ion displacement effects, coordination numbers were calculated from integrated RDFs between phosphate oxygen atoms of lipid headgroups (PE, PG, CL) and either Na^+^ or the positively charged nitrogen atoms of the ionic liquids (see Table S2, Supporting Information). In most systems, Na^+^ retains significant first-shell coordination (coordination number 0.21–0.56). An exception is PG in the Gram-negative membrane-mimicking system (0.04), reflecting the lipid- and composition-dependent accessibility of phosphate groups. In contrast, FIL cations show negligible direct contact (coordination number equal to or close to 0) and accumulate predominantly in the second coordination shell (second coordination number up to 1.14 for [C_14_-Men-Am] at CL in the Gram-positive membrane-mimicking model). This behavior is consistent across Gram-negative and Gram-positive systems and indicates electrostatic screening rather than the competitive displacement of Na^+^ from the phosphate contact shell. The effect is more pronounced for ammonium-based [C_14_-Men-Am] compared to imidazolium-based [C_14_-Men-Im], particularly in cardiolipin-rich environments.

Importantly, these simulations were performed in a simplified monovalent ion environment (Na^+^ only) and do not include divalent cations or lipopolysaccharides; therefore, the observed charge compensation reflects model electrostatic organization rather than full biological ion competition.

### 3.4 Langmuir Monolayer Study of FIL/Lipid System

To experimentally probe the interactions proposed by MD, the interactions in binary FIL–lipid systems mimicking FIL–lipid pairs were investigated, and the results are presented in Figure 12 (π–A isotherms with compression modulus *C_s_* ^–1^–π graphs and BAM images are provided in the Supporting Information, Figures S9–S13).

**Figure 12:**
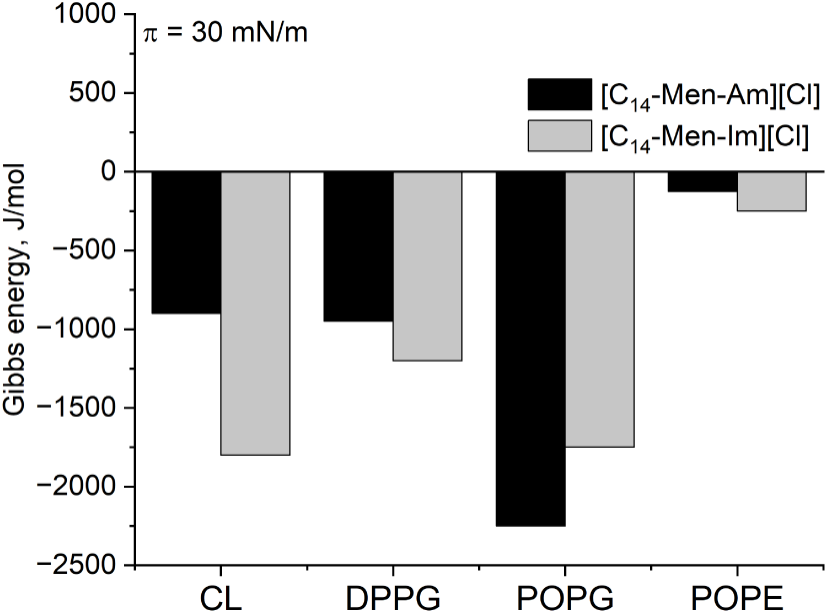
Excess Gibbs free energy of mixing ΔG^exc^ for equimolar lipid/FILs (X_ILs_ = 0.5) systems calculated at 30 mN/m.

Analysis of Figure 12 shows that for mixed FIL/lipid systems with a 1:1 molar ratio, the strongest attractive interactions occur for the PG and CL systems, consistent with the MD simulation results. In contrast, FIL interactions with POPE were the weakest, indicating limited electrostatic affinity toward zwitterionic headgroups. For the imidazolium-based FIL ([C_14_-Men-Im]­[Cl]), the strongest interaction is observed with CL, while for the ammonium analogue ([C_14_-Men-Am]­[Cl]), a more pronounced effect occurs with POPG. A notable observation arises from comparing FIL interactions with POPE (18:1), POPG (18:1), and DPPG (16:0): the key differentiating factor is the nature of the polar headgroup rather than the degree of chain saturation. These results corroborate the MD findings, confirming that electrostatic interactions between the FIL cation and lipid phosphate or glycerol moieties dominate the binding mechanism. Similar conclusions were drawn by Mendonça et al.,^20^ who demonstrated that the cation plays a decisive role in the interaction of imidazolium-based ILs with bacterial-like lipids. They attributed the destabilization of DPPG monolayers by [C_n_mim]Cl to strong electrostatic attraction between the imidazolium cations and negatively charged DPPG headgroups, accompanied by disruption of the close packing of saturated hydrocarbon chains. Analogously, in the present study, incorporation of menthol-based FILs led to a nearly 2-fold reduction in the compression modulus, accompanied by an increase in interaction strength, indicating a greater perturbation of the monolayer mixing thermodynamics and altered interfacial organization, as further supported by microscopic observations. Minor discrepancies relative to the MD data—where [C_14_-Men-Im]­[Cl] showed the strongest affinity for DPPG rather than POPG—can be attributed to system differences: Langmuir monolayers represent binary FIL/lipid mixtures, whereas in MD simulations, the corresponding IL-lipid pairs were analyzed in the environment of other lipids. Nevertheless, the trend is the same and indicates the key role of PG and CL in the interactions of ILs with bacterial lipid membranes.

To sum up, our findings from the Langmuir monolayer technique show that the investigated FILs cause destruction of artificial Gram-positive and Gram-negative bacterial model membranes, but [C_14_-Men-Am]­[Cl] is slightly more efficient. However, as studies on different ILs show, ammonium-based salts can be a little more toxic than imidazole-based ones.^63–67^ Our results indicate that substituting the ammonium cation with an imidazolium core retains antibacterial efficacy and concurrently lowers toxicity. The mode of action of ILs at the membrane level may involve not only changes in lipid packing, resulting from induced hydrocarbon-chain disorder, but also modification of the phospholipid surface anchoring (due to the disruption of interlipid hydrogen bonding or lipid reorientation). As MD simulations showed, both FILs exhibited a shared fundamental mechanism of disrupting artificial bacterial membranes. [C_14_-Men-Am]­[Cl] and [C_14_-Men-Im]­[Cl] were strongly attracted to anionic lipid headgroups (phosphatidylglycerol in both membrane models, and cardiolipin in the Gram-positive membrane-mimicking system), consistent with the notion that cationic amphiphiles target negatively charged bacterial membranes. In all simulations, the FIL cationic heads anchored at the membrane interface (∼15–20 Å from the bilayer center) via electrostatic attraction to phosphate oxygens, while their alkyl tails inserted into the hydrophobic core, spanning the bilayer thickness.

This interfacial binding resulted in the accumulation of FIL cations within the headgroup region and the reorganization of the local ionic environment. Analysis of Na^+^ density profiles and coordination numbers indicates that sodium ions remain present at the membrane interface; however, the presence of FILs modifies their spatial distribution relative to phosphate groups. Rather than direct competitive displacement from the contact shell, FIL cations predominantly accumulate within the interfacial region and contribute to the electrostatic screening of the negatively charged headgroups. Such a redistribution of interfacial charges perturbs the electrostatic balance, stabilizing the membrane. A comparable electrostatic mechanism has been described for other cationic antimicrobials that alter cation–membrane interactions in Gram-negative outer membranes,^68^ although the present simulations were conducted in a simplified monovalent ion model without divalent cations (Ca^2+^/Mg^2+^) or liposaccharides. By modification of charge compensation at the interface, FILs locally alter the membrane ionic environment, a perturbation that likely contributes to membrane destabilization and is consistent with the experimentally observed increase in monolayer fluidity and collapse pressure in FIL-treated films.

Notably, both functionalized salts achieved a similar overall level of antibacterial activity in our experiments, which aligns with the simulations showing that each FIL robustly associates with bacterial lipids and inserts into the bilayer, causing membrane structural disruptions in both Gram-positive and Gram-negative models. Despite these commonalities, our MD results also reveal distinct interaction nuances for the ammonium versus imidazolium FIL, as well as their subtle differences in interactions in Gram-positive vs Gram-negative membranes. [C_14_-Men-Am]­[Cl] showed a pronounced affinity for cardiolipin in the Gram-positive membrane. Its cationic ammonium center preferentially clustered at CL-rich sites, yielding the highest radial distribution peaks with cardiolipin among all of the lipids. This indicates that [C_14_-Men-Am]­[Cl] can target cardiolipin-enriched domains—an important insight since cardiolipin microdomains are known hot spots for bacterial membrane perturbation.^69^ The strong binding of [C_14_-Men-Am] to CL (and to a lesser extent saturated PG) suggests that it embeds into the more tightly packed, rigid regions of the Gram-positive membrane-mimicking system. In contrast, [C_14_-Men-Im]­[Cl] interacted more evenly across anionic lipids and demonstrated a relative preference for unsaturated PG (POPG) in the Gram-negative membrane-mimicking system, despite POPG’s low abundance there. The imidazolium’s delocalized charge and ring structure appear to accommodate the looser packing and higher fluidity of unsaturated lipid regions, whereas the ammonium FIL favored the ordered, saturated lipid environment. These differences in lipid selectivity may underlie subtle variations in how each FIL perturbs artificial membranes: for instance, [C_14_-Men-Am]­[Cl] targeting CL could drive localized domain disruptions in model Gram-positive membranes, while [C_14_-Men-Im] more readily penetrates artificial Gram-negative inner membranes rich in fluid phospholipids. Importantly, both ILs still bound strongly to PG and CL overall, explaining the comparable antibacterial efficacy observed for both membrane models. The simulations thereby provide a molecular rationale for the experimental finding that interactions with CL and POPG were crucial for membrane disintegration and help explain how each FIL can achieve comparable antibacterial effects through slightly different lipid interaction modes. Recall that based on the ΔG^exc^ results for the two-component systems FIL/lipid, the strength of the interactions for [C_14_-Men-Am]­[Cl] was similar for CL and DPPG and stronger for POPG, while for [C_14_-Men-Im]­[Cl] the interactions were similar for CL and POPG and slightly weaker for DPPG. However, these systems were purely model and neglected the influence of the other lipids (which was not omitted in the MD simulations). At the molecular level, the FIL–lipid interactions were dominated by electrostatic attractions rather than specific hydrogen bonds. We detected no persistent hydrogen bonding between the IL cations and lipid headgroups in either membrane model: the closest approach distances (∼4 Å between the IL’s quaternary nitrogen and lipid phosphate oxygen) were characteristic of direct ion–ion or ion–dipole contacts. While the menthol-based substituent of ILs carries an oxygen atom capable of hydrogen bonding, any such interactions (e.g., a transient bridge via a water molecule to a lipid phosphate or glycerol oxygen) were fleeting, occurring only a few percent of the time. Thus, both FILs behave largely as dynamic, surface-bound surfactants—their charged heads interacting transiently and diffusely with anionic sites—and their hydrophobic tails disrupting the lipid bilayer structure. Ultimately, the preferential binding to cardiolipin and PG (abundant in bacterial membranes) over zwitterionic lipids explains the ILs’ selectivity for bacteria over host cells, highlighting a promising route for designing effective and selective antimicrobial agents based on tailored ILs.

## 4 Conclusion and Perspectives

### 4.1 Conclusion

The development of new antimicrobial strategies is urgently needed to limit infections caused by antibiotic-resistant bacteria. This work demonstrates that monoterpene-based functionalized ionic liquids effectively disrupt artificial bacterial membranes through a shared interfacial mechanism driven by the electrostatic attraction to anionic lipid headgroups. Both [C_14_-Men-Am]­[Cl] and [C_14_-Men-Im]­[Cl] readily insert into Gram-positive and Gram-negative membrane-mimicking systems, perturb lipid packing, and induce significant changes in interfacial organization and mixing behavior, as evidenced by thermodynamic calculations and the BAM and AFM observations.

Moreover, replacing the ammonium with an imidazolium cation preserved antibacterial potency while potentially reducing toxicity, highlighting the value of cation tuning in the IL design. MD simulations reveal that cationic anchoring to phosphate groups and subsequent tail insertion into the hydrophobic core drive model membrane perturbation, while lipid selectivity—particularly for cardiolipin and phosphatidylglycerol—enhances activity toward bacterial interfaces. Distinct preferences for saturated versus unsaturated lipid environments offer additional avenues for tuning membrane-specific interactions.

This work highlights the importance of integrating experimental and theoretical approaches to achieve a comprehensive understanding of drug–membrane interactions. While Langmuir monolayers provide thermodynamic parameters such as ΔG^exc^ and reveal macroscopic changes in film organization, they do not allow direct identification of the specific molecular interactions responsible for these effects. By complementing the experiments with MD simulations, we are able to determine which concrete interaction motifs—particularly electrostatic anchoring of the FIL cations to anionic lipid phosphate groups versus only minor, transient hydrogen bonding—underlie the observed nonideality in ΔG^exc^. This combined approach therefore enables a more detailed and mechanistically grounded analysis of how FILs perturb bacterial membrane-mimicking systems, offering insights that cannot be obtained from experimental measurements alone.

Although the studied FILs are not yet in commercial use, antibacterial therapy strategies based on ILs are being widely tested.^70,71^ Further studies should be conducted to evaluate their safety and efficacy under conditions relevant to practical applications, particularly in the context of disinfectant formulations (e.g., medical equipment, hospital surfaces, or controlled topical use). A comprehensive investigation of the physicochemical and biological properties of ILs is required to balance antimicrobial efficacy with minimal side effects. Toxicity toward mammalian cells should also be carefully studied. Nevertheless, as shown in this work, the future of ILs as antibacterial agents is highly promising and opens numerous possibilities for their further development.

### 4.2 Perspectives

The insights gained here support the development of ILs as versatile antibacterial agents with controllable interfacial properties. Future work should evaluate cytotoxicity toward mammalian cells, explore broader structure–property relationships, and integrate ILs into functional surfaces or coatings for biomedical and disinfection applications. With continued optimization, tailored ILs hold strong potential as next-generation tools for combating antibiotic-resistant pathogens in localized and surface-based antimicrobial technologies.

## Supplementary Material



## Notes

BAM textures of Gram-positive and Gram-negative membrane-mimicking monolayers before and after the addition of [C_14_-Men-Am]­[Cl] and [C_14_-Men-Im]­[Cl] at different surface pressures (Figures S1–S2, S10–S13); AFM topography images with height profiles (Figure S3); molecular dynamics analysis of area per molecule vs time (Figure S4) and averaged area per lipid values (Table S1); radial distribution functions between ionic liquid functional groups and lipid headgroup oxygen atoms (Figures S5–S8); coordination numbers derived from RDFs for Na^+^ and ionic liquid cations with phosphate groups (Table S2); π–A isotherms and compressibility modulus plots for CL, DPPG, POPG, and POPE in the absence and presence of ionic liquids (Figure S9) (PDF)
