# Supplementary material for: Functionalized Ionic Liquids at Bacterial Membrane Interfaces: A Multiscale Perspective from Tensiometry, Microscopy, and Molecular Dynamics
Source: J Phys Chem B. 2026 Mar 25;130(14):3888–903. doi: 10.1021/acs.jpcb.5c08635 (PMC13298882; doi:10.1021/acs.jpcb.5c08635)
Supplement: Supplementary file 1 [file jp5c08635_si_001.pdf]

Supplementary Information for

Functionalized Ionic Liquids at Bacterial Membrane Interfaces: A Multiscale Perspective From Tensiometry, Microscopy, And Molecular Dynamics

Anita Wnętrzak[a*], Joanna Feder-Kubis[b,c], Anna Chachaj-Brekiesz[a], Patrycja Dynarowicz-Latka[a] , Jan Kobierski[d*]

[a] *Faculty of Chemistry, Jagiellonian University, Gronostajowa 2, Kraków, 30-387, Poland*
[b] *Faculty of Chemistry, Wrocław University of Science and Technology, Wybrzeże Wyspiańskiego 27, Wrocław, 50-370, Poland*
[c] *Department of Inorganic Chemistry, Technische Universität Dresden, 01069 Dresden, Germany*
[d] *Jagiellonian University Medical College, Faculty of Pharmacy, Department of Pharmaceutical Biophysics, Medyczna 9, 30-688 Kraków, Poland*
[e] *Faculty of Chemistry, University of Bialystok, Ciolkowskiego 1K, 15-245 Bialystok, Poland*

*anita.wnetrzak@uj.edu.pl, jan.kobierski@uj.edu.pl

Table of contents:

Figure S1. Textures of investigated films mimicking membranes of Gram-positive bacteria before and after ILs addition (X_{IL}=0.25) visualized with BAM at different surface pressure (BAM images show monolayer fragments of 720 μm × 400 μm)………………………………………………………………... S2
Figure S2. Textures of investigated films mimicking membranes of Gram-positive bacteria before and after ILs addition visualized with BAM at different surface pressure (BAM images show monolayer fragments of 720 μm × 400 μm)………………………………………………………………………..S3
Figure S3. AFM topography images (5 μm x 5 μm) together with profiles extracted along white lines (1 and 2): A) system mimicking Gram-positive bacterial membrane B) system mixed with [C_{14}-Men-Am][Cl]; C) system mixed with [C_{14}-Men-Im][Cl]…………………………..............................S3
Figure S4. Area per molecule as a function of time simulated in molecular dynamics simulations for Gram-negative (A and B) and Gram-positive (C and D) membrane systems. Ionic liquids were added at a molar fraction of 0.24: [C14-Men-Am][Cl] in systems A and C, and [C14-Men-Im][Cl] in systems B and D……………………………………………………………………………………...S4
Table S1. Area per molecule values obtained from molecular dynamics simulations at the corresponding surface pressures, averaged over the final 50 ns of simulated time. Results are shown for Gram-negative and Gram-positive membrane models in the presence of [C_{14}-Men-Im][Cl] and [C_{14}-Men-Am][Cl], each introduced at a molar fraction of 0.25………………………………………S4
Figure S5. Radial distribution functions for pairs involving either the nitrogen atom (top panel) or the oxygen atom (bottom panel) of [C_{14}-Men-Am][Cl], and either the non-esterified phosphate oxygen (left panel) or the terminal glycerol oxygen (right panel) of phosphatidylglycerols in the Gram-negative membrane model………………………………………………………...…………...…………..S5
Figure S6. Radial distribution functions for pairs involving either the nitrogen N1 atom (top panel), the nitrogen N2 atom (middle panel) or the oxygen atom (bottom panel) of [C_{14}-Men-Im][Cl], and either the non-esterified phosphate oxygen (left panel) or the terminal glycerol oxygen

(right panel) of phosphatidylglycerols in the Gram-negative membrane model. ……………………………………..……………………………………………………….…S6
Figure S7. Radial distribution functions for pairs involving either the nitrogen atom (top panel) or the oxygen atom (bottom panel) of [C_{14}-Men-Am][Cl], and either the non-esterified phosphate oxygen (left panel) or the terminal glycerol oxygen (right panel) of cardiolipin phosphatidylglycerols in the Gram-negative membrane model…………………………………………………………………………..S7
Figure S8. Radial distribution functions for pairs involving either the nitrogen N1 atom (top panel), the nitrogen N2 atom (middle panel) or the oxygen atom (bottom panel) of [C_{14}-Men-Im][Cl], and either the non-esterified phosphate oxygen (left panel) or the terminal glycerol oxygen (right panel) of phosphatidylglycerols or cardiolipin in the Gram-positive membrane model……………………………………………………………………………………………..S8
Figure S9. π-A isotherms of the investigated lipid films (CL, DPPG, POPG and POPE) without and in the presence of ILs (X=0.25): [C_{14}-Men-Am][Cl] (A-D) and [C_{14}-Men-Im][Cl] (E-H) . Insets: compressibility modulus Cs^{-1}- π dependencies……………………………………………...………S9
Figure S10. Textures of investigated lipid films of CL before and after ILs addition in proportion of X=0.5 visualized with BAM at different surface pressure (BAM images show monolayer fragments of 720 μm × 400 μm)……………………………………………………………………………………S10
Figure S11. Textures of investigated lipid films of DPPG before and after ILs addition in proportion of X=0.5 visualized with BAM at different surface pressure (BAM images show monolayer fragments of 720 μm × 400 μm)……………………………………………...S11
Figure S12. Textures of investigated lipid films of POPG before and after ILs addition in proportion of X=0.5 visualized with BAM at different surface pressure (BAM images show monolayer fragments of 720 μm × 400 μm)……………………………………………………………………………………S12
Figure S13. Textures of investigated lipid films of POPE before and after ILs addition in proportion of X=0.5 visualized with BAM at different surface pressure (BAM images show monolayer fragments of 720 μm × 400 μm)…………………………………………………………………………..……...S13

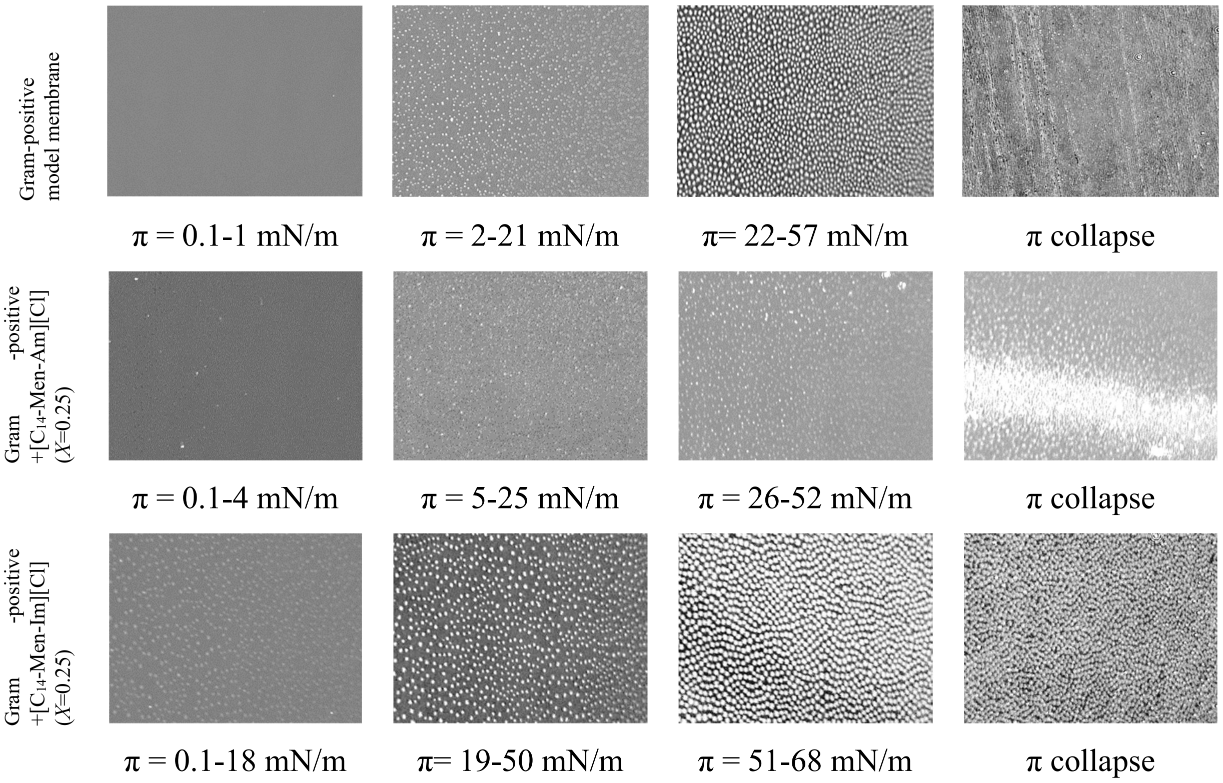

Figure S1. Textures of investigated films mimicking membranes of Gram-positive bacteria before and after ILs addition (X_{IL}=0.25) visualized with BAM at different surface pressure (BAM images show monolayer fragments of 720 μm × 400 μm).

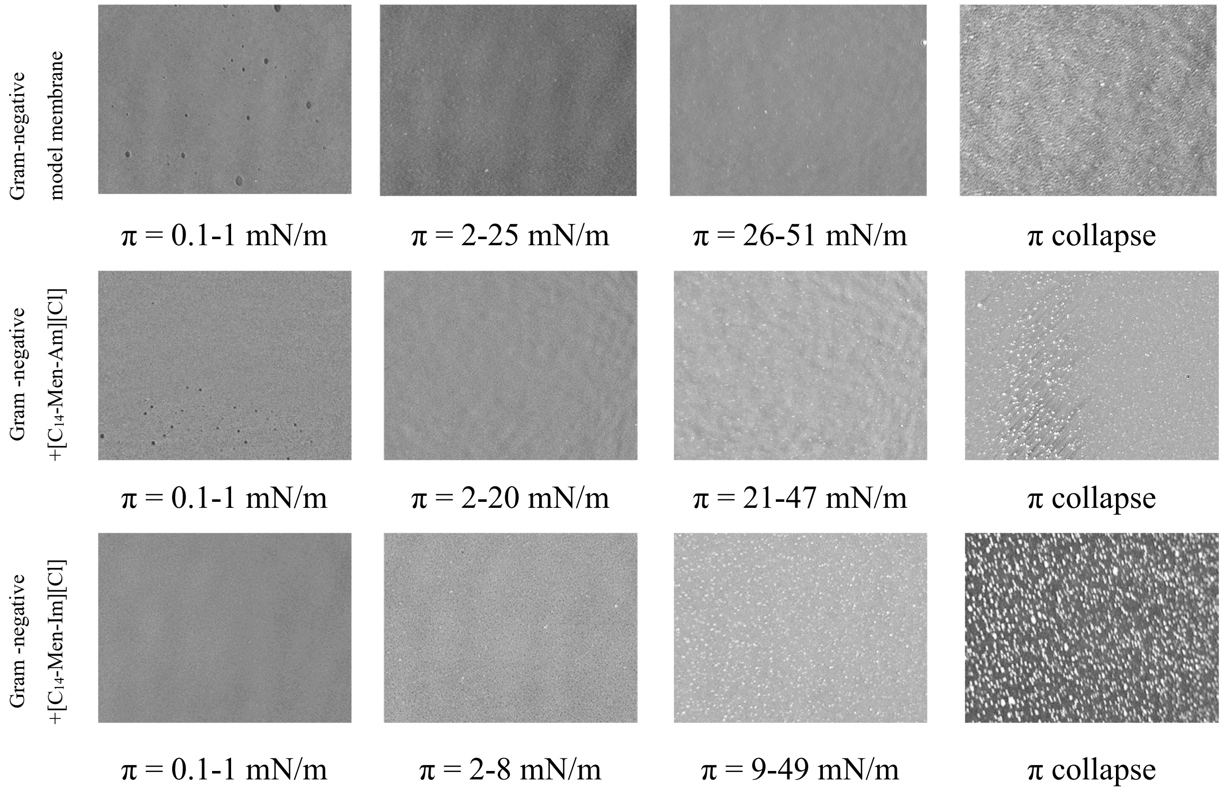

Figure S2. Textures of investigated films mimicking membranes of Gram-positive bacteria before and after ILs addition visualized with BAM at different surface pressure (BAM images show monolayer fragments of 720 μm × 400 μm).

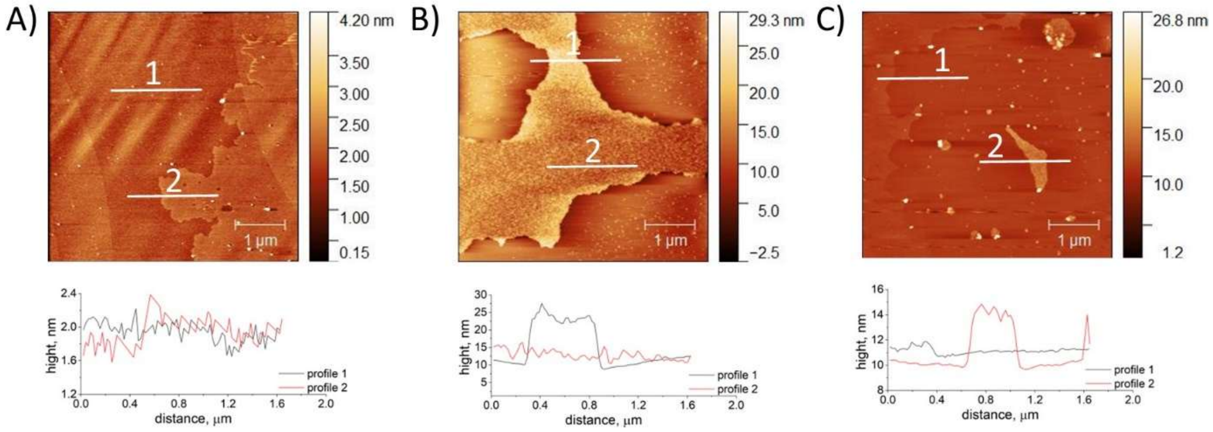

Figure S3. AFM topography images (5 μm x 5 μm) together with profiles extracted along white lines (1 and 2): A) system mimicking Gram-positive bacterial membrane B) system mixed with [C_{14}-Men-Am][Cl]; C) system mixed with [C_{14}-Men-Im][Cl];

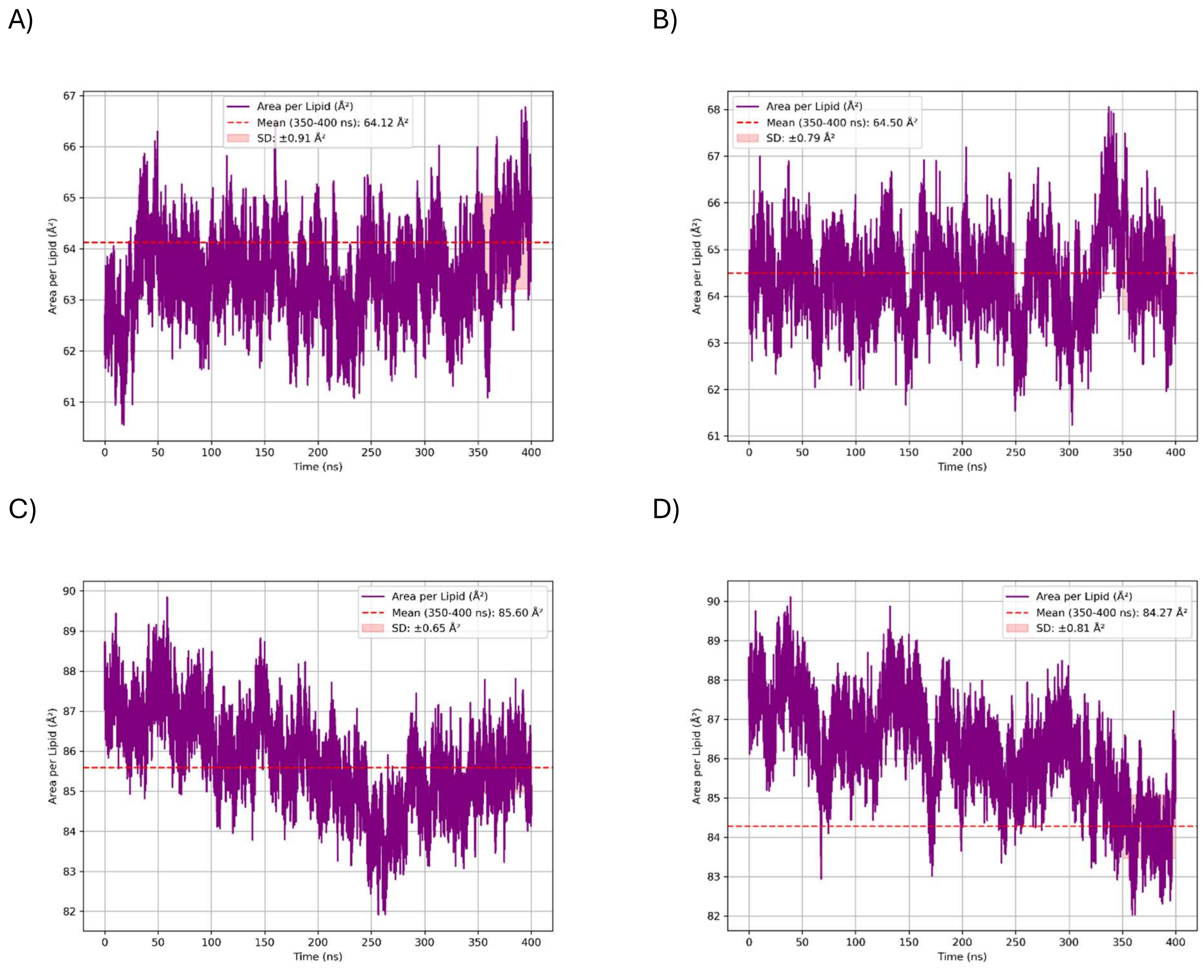

Figure S4. Area per molecule as a function of time simulated in molecular dynamics simulations for Gram-negative (A and B) and Gram-positive (C and D) membrane systems. Ionic liquids were added at a molar fraction of 0.24: [C14-Men-Am][Cl] in systems A and C, and [C14-Men-Im][Cl] in systems B and D.

Table S1. Area per molecule values obtained from molecular dynamics simulations at the corresponding surface pressures, averaged over the final 50 ns of simulated time. Results are shown for Gram-negative and Gram-positive membrane models in the presence of [C_{14}-Men-Im][Cl] and [C_{14}-Men-Am][Cl], each introduced at a molar fraction of 0.25.

Bacteria membrane model + [X_{IL} = 0.25]	Surface pressure [mN/m]	Area per lipid [Å^2]	
		Mean	SD
Gram-negative + [C_{14}-Men-Am][Cl]	34.0	64.12	0.91
Gram-negative + [C_{14}-Men-Im][Cl]	33.6	64.50	0.79
Gram-positive + [C_{14}-Men-Am][Cl]	33.3	85.60	0.65
Gram-positive + [C_{14}-Men-Im][Cl]	33.6	84.27	0.81

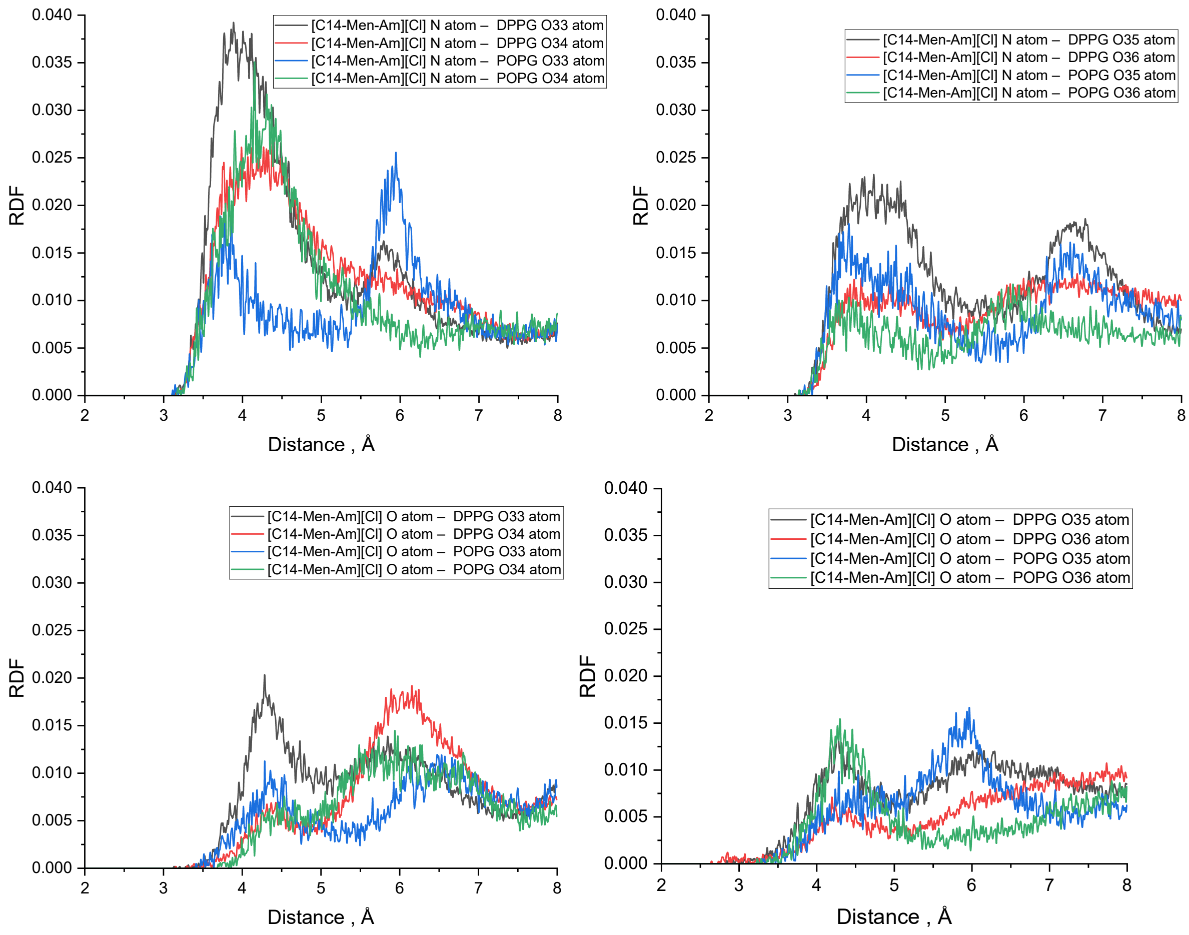

Figure S5. Radial distribution functions for pairs involving either the nitrogen atom (top panel) or the oxygen atom (bottom panel) of [C_{14}-Men-Am][Cl], and either the non-esterified phosphate oxygen (left panel) or the terminal glycerol oxygen (right panel) of phosphatidylglycerols in the Gram-negative membrane model.

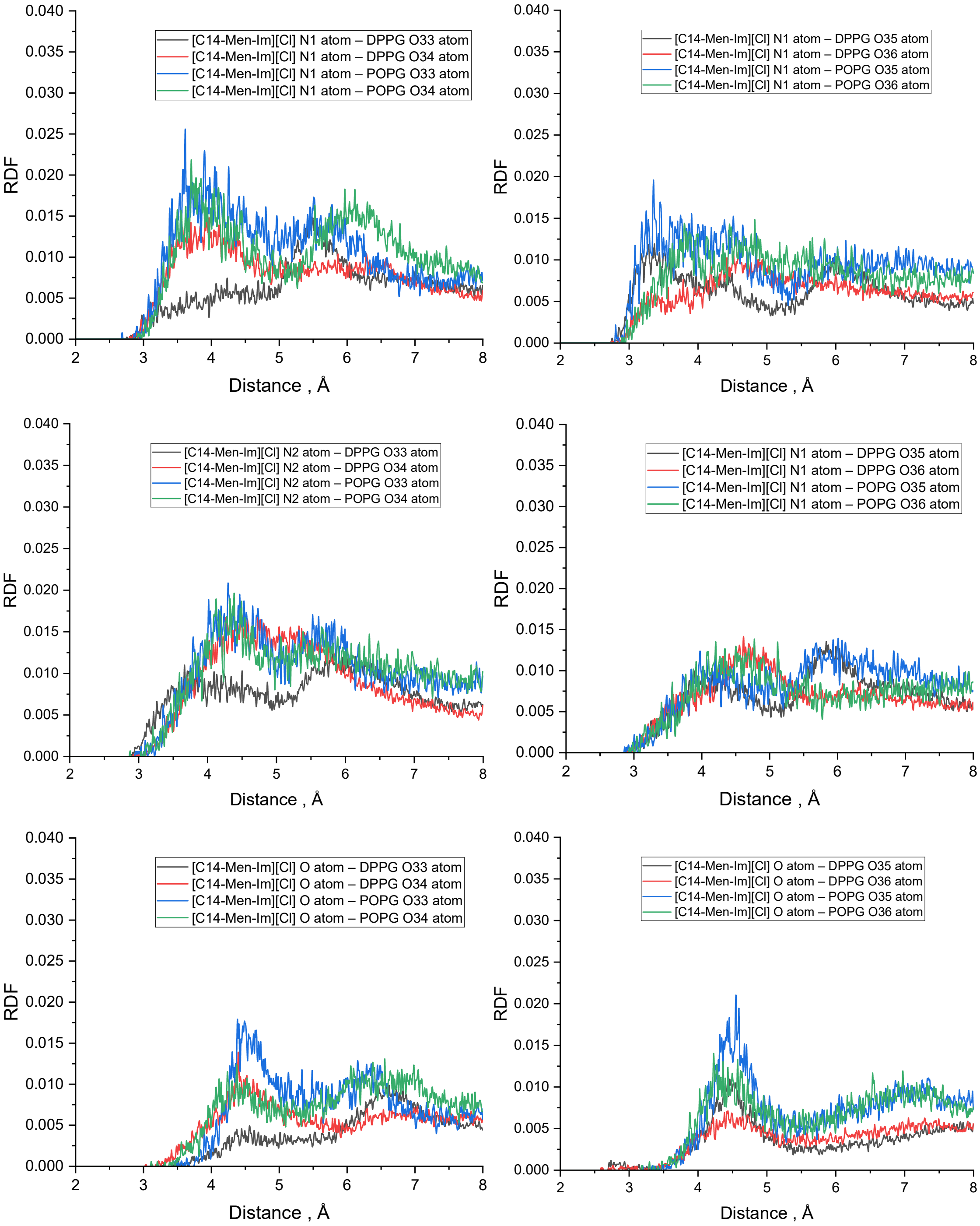

Figure S6. Radial distribution functions for pairs involving either the nitrogen N1 atom (top panel), the nitrogen N2 atom (middle panel) or the oxygen atom (bottom panel) of [C_{14}-Men-Im][Cl], and either the non-esterified phosphate oxygen (left panel) or the terminal glycerol oxygen (right panel) of phosphatidylglycerols in the Gram-negative membrane model.

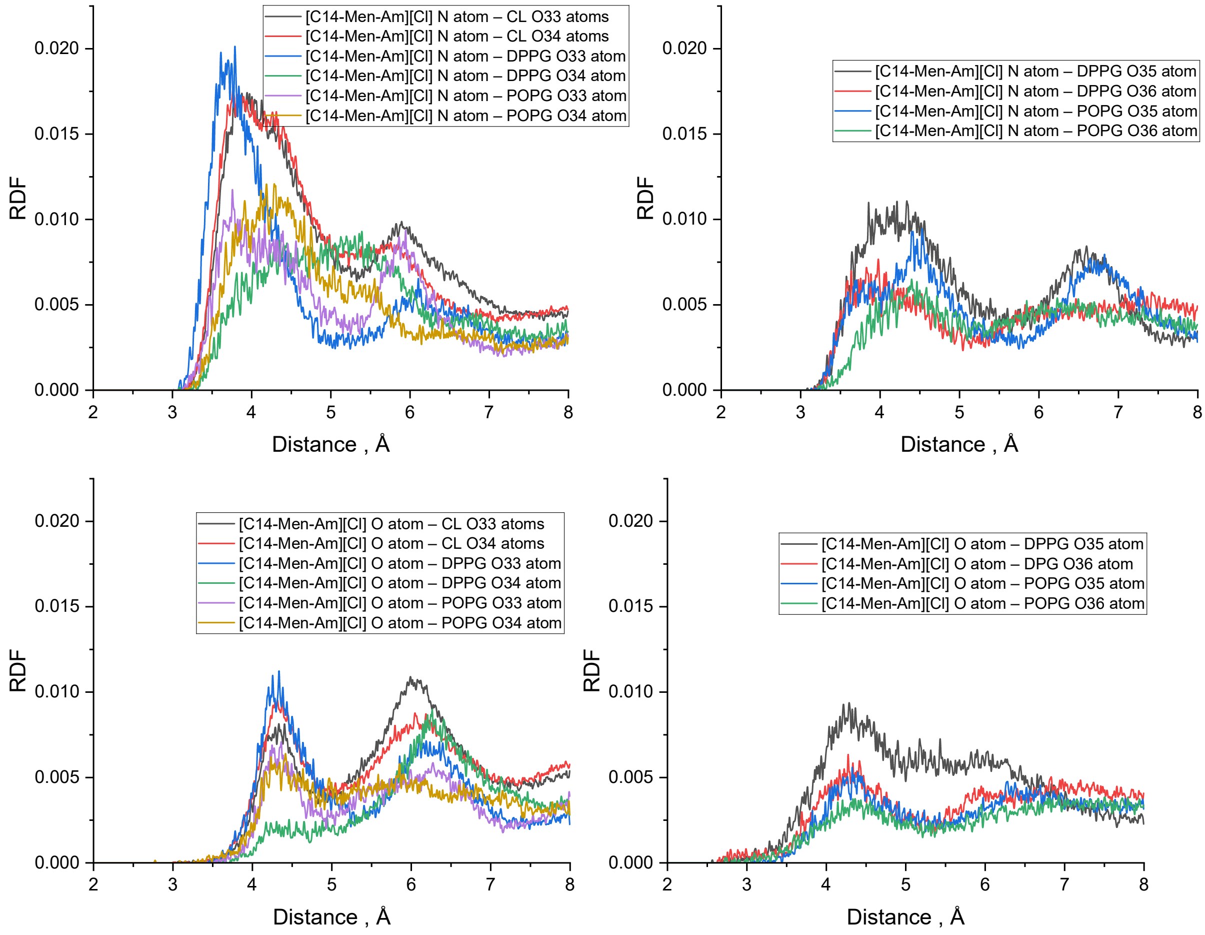

Figure S7. Radial distribution functions for pairs involving either the nitrogen atom (top panel) or the oxygen atom (bottom panel) of [C_{14}-Men-Am][Cl], and either the non-esterified phosphate oxygen (left panel) or the terminal glycerol oxygen (right panel) of cardiolipin phosphatidylglycerols in the Gram-negative membrane model.

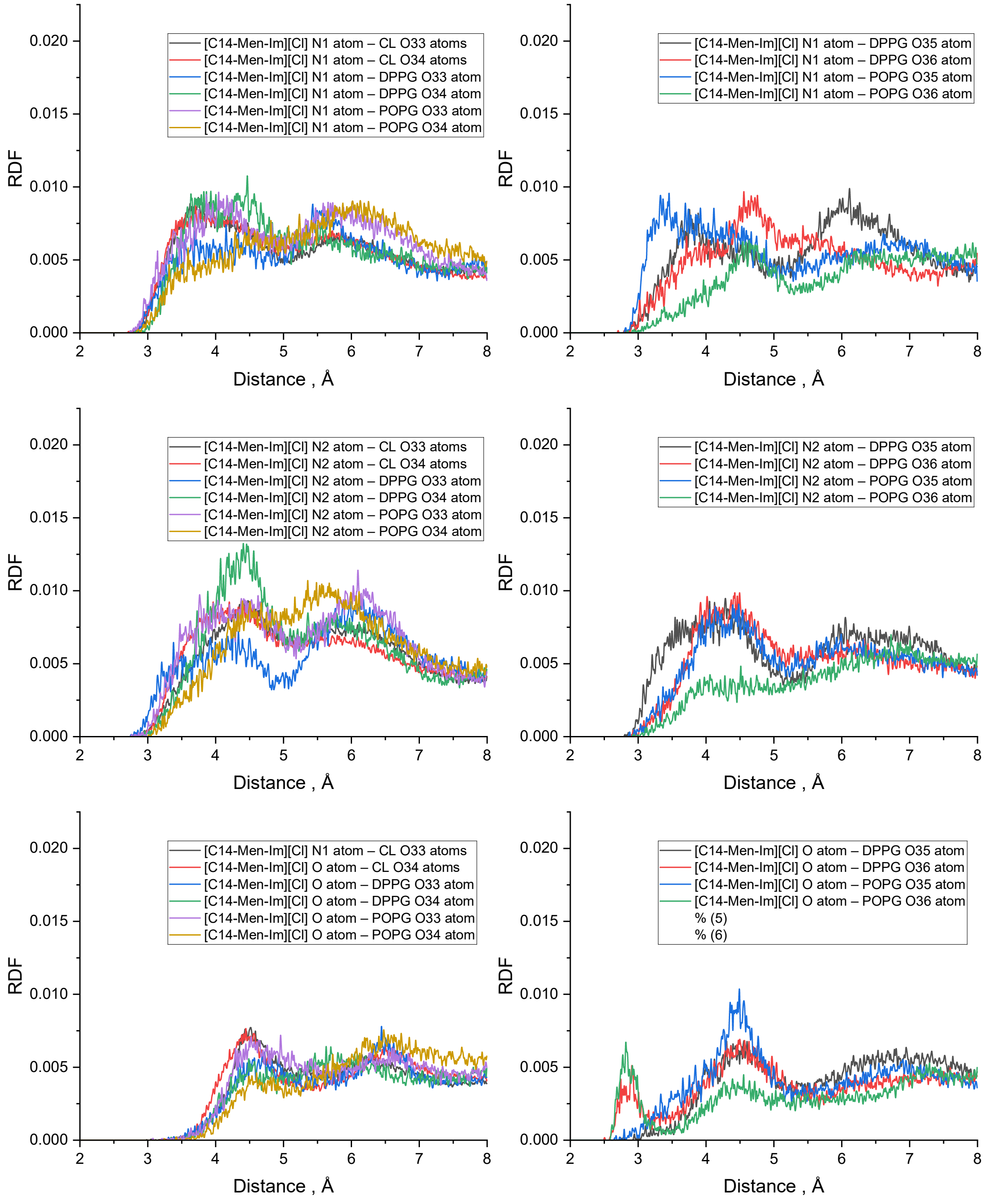

Figure S8. Radial distribution functions for pairs involving either the nitrogen N1 atom (top panel), the nitrogen N2 atom (middle panel) or the oxygen atom (bottom panel) of [C_{14}-Men-Im][Cl], and either the non-esterified phosphate oxygen (left panel) or the terminal glycerol oxygen (right panel) of phosphatidylglycerols or cardiolipin in the Gram-positive membrane model.

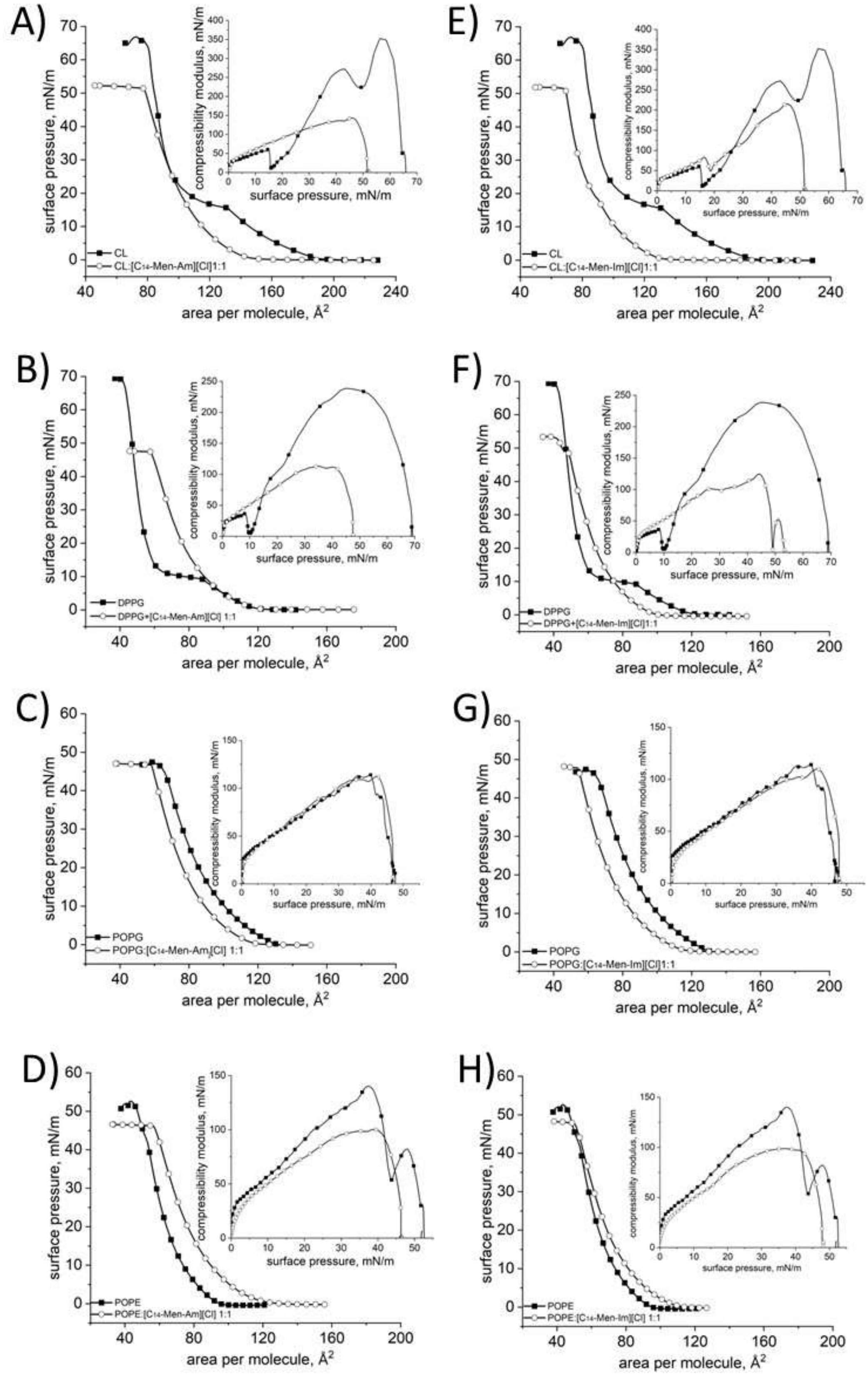

Figure S9. π-A isotherms of the investigated lipid films (CL, DPPG, POPG and POPE) without and in the presence of ILs (X=0.25): [C_{14}-Men-Am][Cl] (A-D) and [C_{14}-Men-Im][Cl] (E-H) . Insets: compressibility modulus Cs^{-1}- π dependencies.

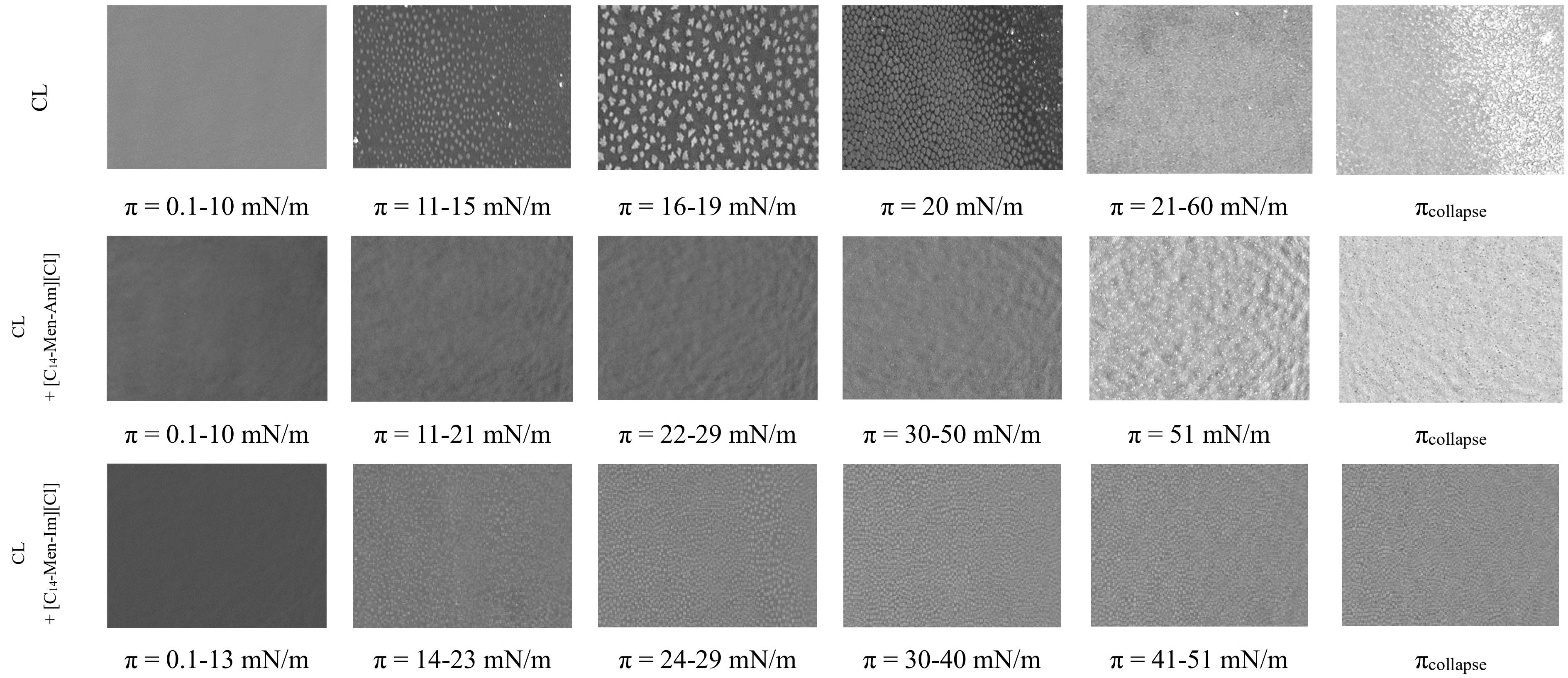

Figure S10. Textures of investigated lipid films of CL before and after ILs addition in proportion of X=0.5 visualized with BAM at different surface pressure (BAM images show monolayer fragments of 720 µm × 400 µm).

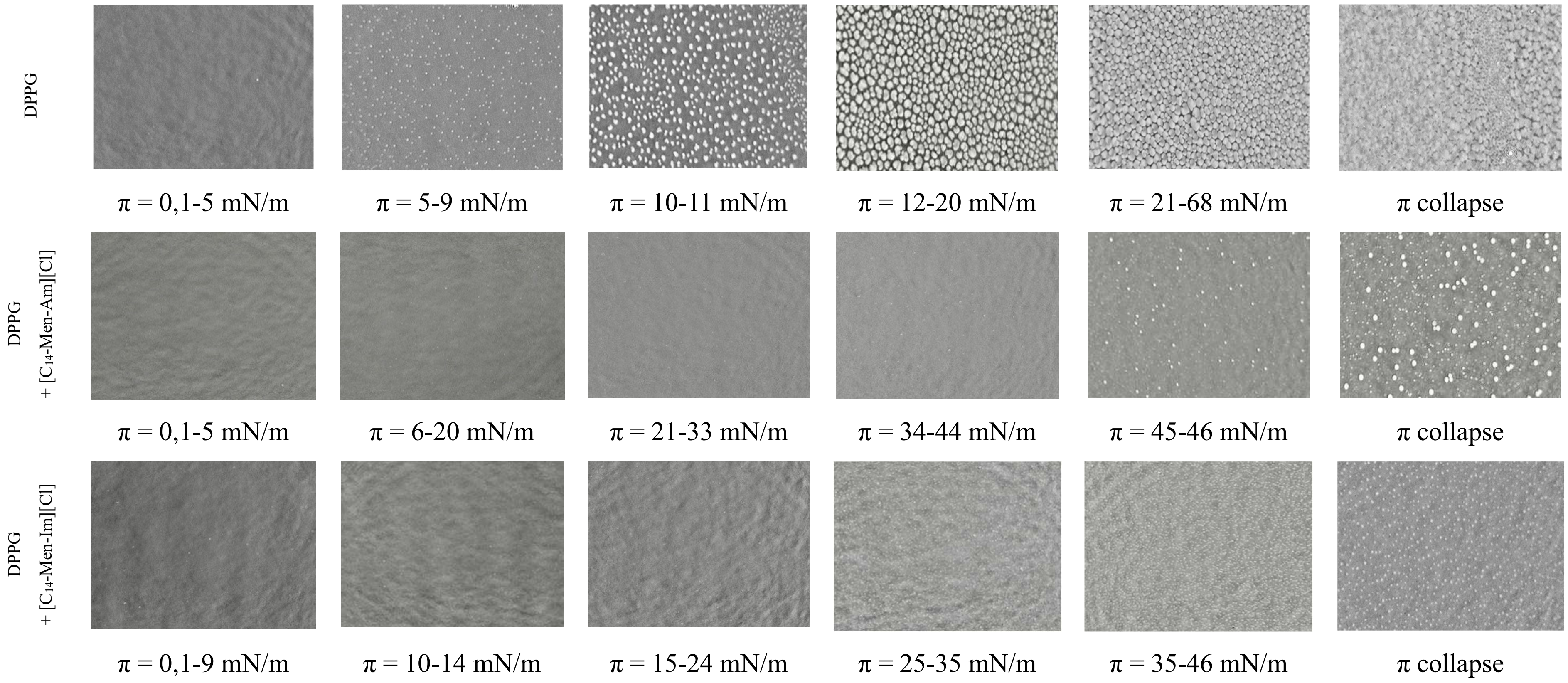

Figure S11. Textures of investigated lipid films of DPPG before and after ILs addition in proportion of X=0.5 visualized with BAM at different surface pressure (BAM images show monolayer fragments of 720 µm × 400 µm).

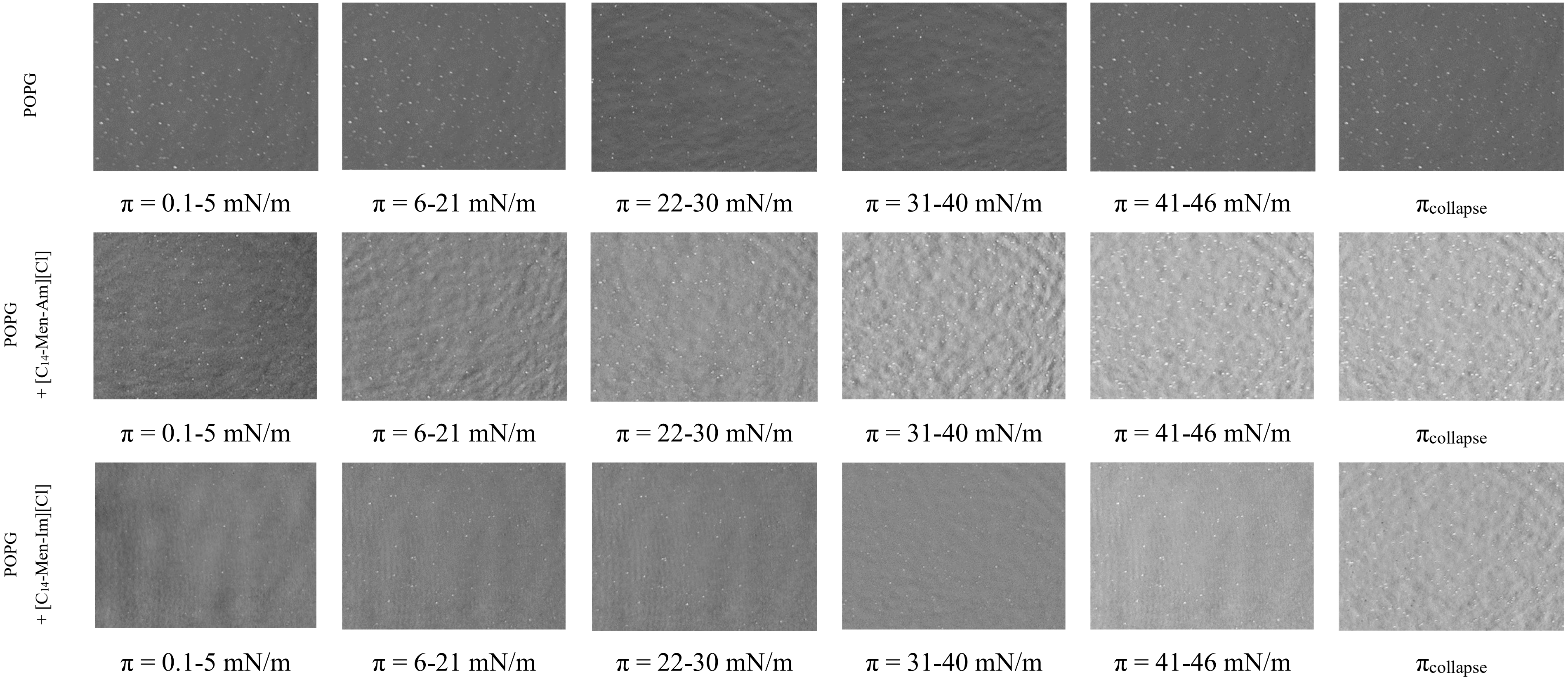

Figure S12. Textures of investigated lipid films of POPG before and after ILs addition in proportion of X=0.5 visualized with BAM at different surface pressure (BAM images show monolayer fragments of 720 µm × 400 µm).

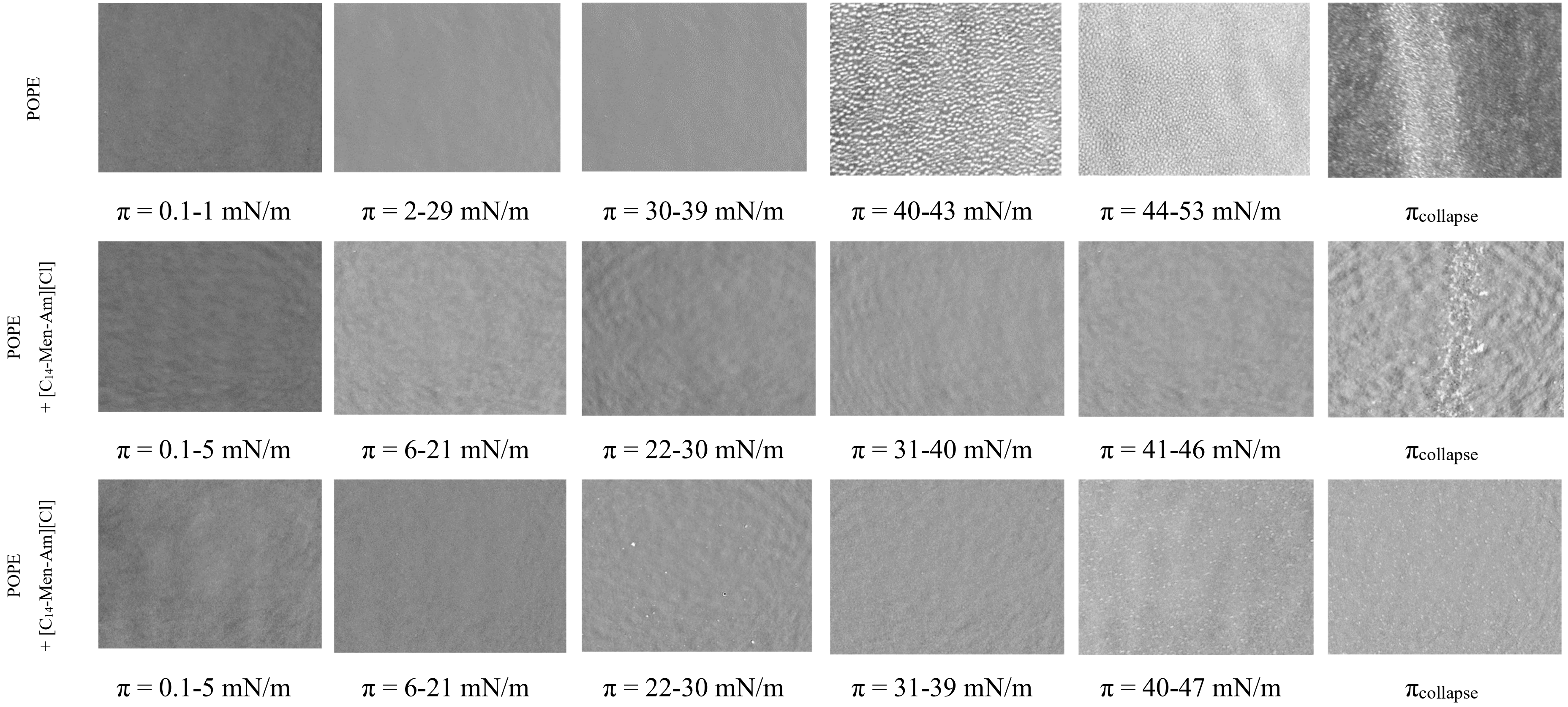

Figure S13. Textures of investigated lipid films of POPE before and after ILs addition in proportion of X=0.5 visualized with BAM at different surface pressure (BAM images show monolayer fragments of 720 μm × 400 μm
